# Healthcare under fire: quantifying the impact of violence on medical services in facilities supported by International Medical Corps in three prefectures of Central African Republic, 2016–2020

**DOI:** 10.1186/s13031-025-00686-w

**Published:** 2025-08-05

**Authors:** Natalya Kostandova, Jennifer OKeeffe, Audrey Mahieu, Blaise Bienvenu Ali, Christian Mulamba, Pierre Somsé, Odilon Guesset Bingou IV, Sebastien Dackpa, Gerard Mbonimpa, Thierry Fikiri, Larissa Fast, Leonard Rubenstein

**Affiliations:** 1 International Medical Corps, Washington, DC USA; 2 Department of Epidemiology, Johns Hopkins Bloomberg School of Public Health, Baltimore, MD USA; 3 Department of International Health, Johns Hopkins Bloomberg School of Public Health, Baltimore, MD USA; 4 Geneva Centre of Humanitarian Studies, University of Geneva, Geneva, Switzerland; 5 Institut Centrafricain des Statistiques et des Etudes Economiques et Sociales, Bangui, Central African Republic; 6 International Medical Corps, Bangui, Central African Republic; 7 Ministère de la Santé et de la Population, Bangui, Central African Republic; 8 Humanitarian and Conflict Response Institute, University of Manchester, Manchester, UK; 9 Present Address: International Medical Corps, Abuja, Nigeria

**Keywords:** Attacks on healthcare, Central African Republic, Armed conflicts, Healthcare system, Observational studies

## Abstract

**Background:**

Attacks on healthcare in the Central African Republic (CAR) are widespread, under-reported, and inadequately addressed. This study examined the impact of attacks on healthcare in three conflict-affected prefectures—Ouaka, Haute-Kotto, and Vakaga—between 2016 and 2020, assessing both immediate and longer-term disruptions.

**Methods:**

Disruptions at primary and referral facilities were evaluated using program data from International Medical Corps (IMC) and attack data from IMC, key informants, secondary datasets, and local media. Key indicators—outpatient consultations, first antenatal care visits (ANC1), facility deliveries, measles vaccination (MCV1), and hospitalizations—were analyzed using visual trend analysis, estimation of immediate change, and interrupted time series (ITS). Survival analysis assessed the association between attacks and time to the first reported measles case during a nationwide outbreak.

**Results:**

A total of 127 individual attacks were identified over five years, primarily from IMC security reports. The most common incidents were asset removal or attempted removal (27%), threat (18%), and pillage (17%). At least one form of physical or sexual violence was documented in 23.2% of attacks, with 13 instances of murder. Visual analysis showed three impact patterns: facility closure, disruption of specific services, and minimal or short-term changes. Immediate changes also varied, with service changes ranging from − 100 to 655%. In ITS analysis four of eight facilities showed > 25% deficit in outpatient consultations while three showed > 25% surplus. Survival analysis demonstrated significant difference in time to first measles case between attacked and facilities without attacks (*p* < 0.001), though findings are limited by small sample size. Overall, maternal health services had fewer fluctuations while vaccination services ceased altogether in some facilities.

**Conclusions:**

This study shows improved identification and profiling of attacks is possible in low-resource settings and presents several approaches to quantify their impact. It also highlights challenges conducting analysis with significant limitations in data quality and availability. Findings reinforce the urgent need for systematic data collection, real-time monitoring, tailored, context-specific mitigation strategies, and support to local actors who maintain services when external support is limited. Future research should build on these findings to provide more effective protection, mitigation, and recovery strategies for healthcare systems in conflict zones.

**Supplementary Information:**

The online version contains supplementary material available at 10.1186/s13031-025-00686-w.

## Background

Attacks on healthcare in armed conflicts pose a critical threat to health and well-being of populations living in conflict-affected countries. These attacks often undermine healthcare delivery and limit access to essential services for already vulnerable populations [1]. The extent and duration of service disruptions in particular contexts remain poorly understood. Increased knowledge of these effects could contribute to efforts to lessen the impacts of the violence and mitigate its effects.

In the past decade, qualitative studies have assessed how attacks in armed conflicts impact healthcare workers, services, outcomes, and health system capacity [2–8]. While these studies provide valuable insights, they are limited in assessing the attacks’ differential effects on particular services and populations, as well as the duration of impacts. Many have addressed short-term outcomes of attacks [15] and healthcare worker experiences [16–19]. A few quantitative studies have aimed to measure the long- term impact of attacks [13, 14, 20] the majority of which have been conducted in the Middle East Region. Quantitative studies in the African region have been conducted in Burkina Faso [9], Nigeria [11] and Uganda [12] and have primarily focused on the impact on maternal health services.

The paucity of quantitative studies on impacts of violence against healthcare is likely a product of the difficulty of accessing relevant data on service provisions and utilization after attacks due to insecurity, communication barriers, and policies limiting information sharing [1]. In many conflict-affected countries, particularly in West and Central Africa, attacks on healthcare, including pillaging, threats to staff, and restricted access, are so common, they often go unreported, leading to gaps in data [1]. Many countries lack reliable reporting systems, and fear of reprisals further deters reporting. When attacks are reported, response and support may be minimal or absent [6].

This study directly addresses several gaps identified in previous quantitative work. With few exceptions [20], interrupted time series (ITS) analysis or survival analysis have rarely been applied to examine association between attacks and time-to-event outcomes such as disease occurrence following attacks. Similarly, outpatient consultations have seldom been analyzed in this context. Effects on non-maternal health services, including vaccination uptake, hospitalizations, and cases of epidemic-prone diseases, have been rarely quantified. By applying multiple analytical methods and examining a broad range of service indicators, this study contributes new evidence to an under-researched area and offers a more comprehensive understanding of how attacks affect healthcare delivery in fragile and conflict-affected settings and how these effects can be quantified. Few studies have been conducted in neglected crises and to our knowledge, no research has attempted to quantify the impact of attacks against healthcare in the Central African Republic (CAR), a country that has experienced sustained violence against healthcare for years [1, 21, 22].

CAR is one of the poorest countries in the world, ranking 191 of 193 countries on the United Nations Human Development Index [23], with the 5th highest maternal and infant mortality in the world [24]. Of a population of 6.1 million, over one million people are displaced and over three million need humanitarian assistance [25]. CAR has 0.21 physicians per 10,000 people, significantly below the recommended 25 per 10,000 needed for adequate primary healthcare coverage [26]. The country has been embroiled in civil war for more than a decade that has been characterized by extensive violence inflicted on healthcare including killings, physical and sexual assault, abductions, arson, shelling, pillage, occupations, and verbal threats [6].

The health system in CAR is severely under-resourced and heavily impacted by ongoing conflict and instability [24]. It is structured across three levels—primary, secondary, and tertiary care—but access remains limited, especially in rural and conflict-affected areas [27]. The majority of functional health services are supported by international and non-governmental organizations, with significant gaps in infrastructure, workforce, and medical supplies. Public health financing is low, and health information systems are weak, further complicating service delivery and planning. Persistent insecurity has disrupted routine services and contributed to poor health outcomes across the country [27].

The study took place in three rural prefectures- Ouaka, Haute-Kotto, and Vakaga, selected for their significant presence of armed groups, high population displacement, and prevalence of attacks on civilians and health facilities [6]. This paper presents the quantitative component of a mixed-methods study examining the impact of attacks on healthcare in CAR. The study analyzes specific attacks and secondary data from healthcare facilities supported by the Non-Governmental Organization (NGO) International Medical Corps (IMC) to quantify both the short- and long-term effects.

Using key informant interviews, the qualitative component [6] revealed that attacks were more widespread than reported, leading to extended facility closures, significant supply losses, and disproportionate harm to vulnerable groups including children under five, the elderly, people living with disabilities, people with chronic illness, and displaced individuals. Healthcare workers suffered psychological trauma and moral injury from repeated attacks and inability to provide adequate care. Mitigation efforts varied, and were largely reliant on community initiatives [6].

The study was part of the *Researching the Impact of Attacks on Healthcare* project [28] and was a collaboration between IMC, the Johns Hopkins University Bloomberg School of Public Health, Institut Centrafricain des Statistiques et des Etudes Economiques et Sociales (ICASEES), the Ministry of Health and Population (MSP), and the Geneva Centre of Humanitarian Studies at the University of Geneva.

## Methods

### Study design

The study presents the quantitative component of a mixed-methods project that assessed the long-term impact of attacks on healthcare in CAR. The quantitative component focuses on evaluating service disruptions using data on identified attacks and corresponding health facility data. It employed multiple analytical approaches to examine both immediate and longer-term changes in key healthcare utilization indicators following attacks on healthcare facilities to assess their impact.

### Study setting and period

**Fig. 1:**
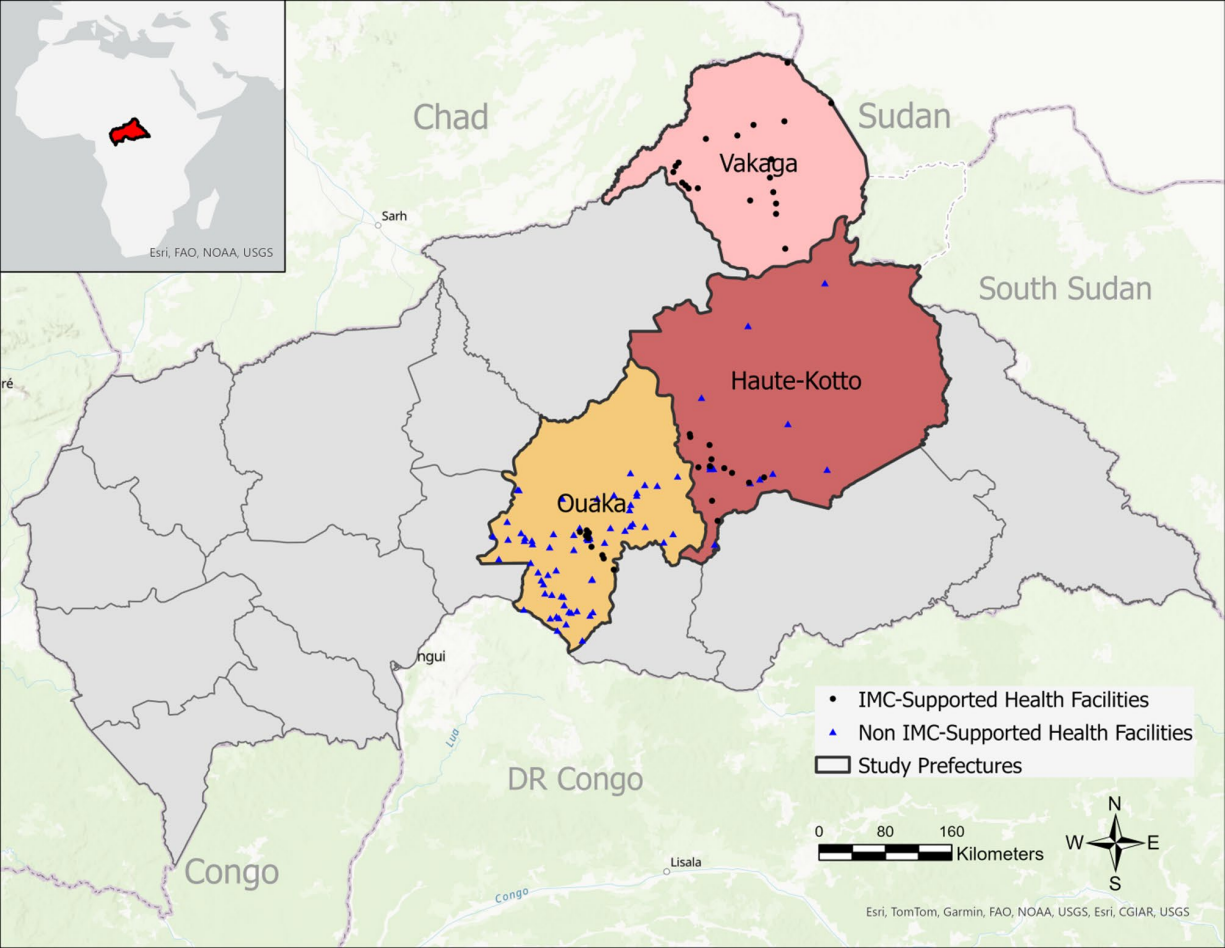
Map of prefectures included in the study, with locations of health facilities supported or operated by International Medical Corps between 2016–2020 in Central African Republic. The facilities presented are those supported in 2019; there was some variability in support coverage over the period of 2016–2020

This analysis includes catchment areas of primary, secondary, and tertiary health facilities (static and mobile) supported by IMC in Haute-Kotto, Ouaka, and Vakaga, from January 2016 to December 2020 (Fig. 1). Across the prefectures, an estimated 46% of facilities are not fully functional due to gaps in financing, infrastructure, personnel, supplies, and medications [27]. Despite the gaps, external financing and support for facilities are only provided for 46% of facilities in the prefectures. In addition to IMC, the level of support and coverage provided by various NGOs to health facilities varied by prefecture and over time. While many international and national NGOs were active in Ouaka and Haute-Kotto, support in Vakaga was more limited, with only the World Health Organization, International Committee of the Red Cross, and Médecins Sans Frontières providing support to health services intermittently between 2016 and 2020.

### Definition of attack on healthcare

We defined an attack on healthcare as “any act of threat, obstruction, physical or verbal violence against health care workers, patients, health facilities or medical transport”, consistent with the World Health Organization (WHO), and extending to personnel on duty, patients, facilities, or transports protected under International Humanitarian Law (IHL) during conflict [29]. Data on attacks on healthcare were derived from both primary and secondary sources.

### Data sources

#### Primary data of attacks on healthcare

Key informant interviews were conducted in French, English, or Sango, depending on interviewee preference, over phone or using encrypted calling software (Skype and Zoom), in three phases (May-June 2022, December 2022-January 2023, and June-August 2023). Participants were selected purposively based on presumed knowledge of attacks on healthcare in the targeted areas between January 2016 and December 2020, and included administrative authorities, frontline health personnel, NGO staff, and regional and district-level health authorities from the MSP.

Interviews solicited attack details (date, location, type, and description), perceived impact on healthcare facilities, services, demand, and utilization, mitigation measures, and any contextual factors potentially affecting healthcare service and utilization. As the study focused on impact of attacks rather than accountability under IHL, key informants were not asked to identify perpetrators, though some did provide this information.

A total of 41 key informants were interviewed, with 36 included in the study. Excluded interviews involved attacks outside the study area or period. Additional details on participant profiles, training, interview guide, and interview processes are available in the complementary publication, which presents qualitative analysis of the key informant interviews [6].

#### Secondary data of attacks on healthcare

Secondary data covering the study period from January 2016 to December 2020 came from four separate sources: (1) Insecurity Insight, (2) IMC, (3) Radio Ndeke Luka, and (4) an anonymous organization.

The Insecurity Insight dataset is publicly available [22] and provided details on attacks including dates, descriptions, facility administrative level, approximate geographical location, and, where available, perpetrator profiles. The anonymous organization shared a dataset with similar variables.

IMC security and donor reports often contained comprehensive narratives on attacks, including dates, descriptions, immediate and long-term effects on IMC operations, and mitigation strategies. The reports also provided broader context of the conflict in targeted areas, including non-healthcare related violence, population displacement, political activities (e.g., elections, visits of political figures in the area, peace negotiations), and other factors potentially affecting healthcare access. Finally, we exhaustively reviewed articles published by Radio Ndeke Luka [30] a national radio and publishing platform, extracting data for reported attacks in the study area and period.

We excluded data on attacks outside the study period or area. If an attack location was unclear in other sources, we confirmed its occurrence in the study area with IMC. When details were uncertain, we requested additional information from Insecurity Insight and triangulated this information. Most of this additional information came from public sources, such as press releases and news stories; for a select few attacks, sources were confidential, with Insecurity Insight providing only general, de-identified information.

#### Health facility data

We collected weekly secondary data from IMC supported facilities on health services including number of: outpatient consultations, first antenatal care (ANC1) visits, doses of measles containing vaccine (MCV1) administered to children under one year old, health facility deliveries, and hospitalizations. Data were stored and extracted from the cloud-based DHIS2 reporting platform. We aggregated DHIS2 data by month.

### Data analysis

#### Identification, triangulation, and profiling of attacks on healthcare

We triangulated primary and secondary data sources to deduplicate attacks on healthcare, or complete missing information such as exact attack dates, from interviews. The final dataset included all attacks mentioned at least once in the primary and secondary datasets, with no distinction made whether an attack was mentioned in a single dataset or in multiple. For each attack, the final database included: Prefecture, location type (health facility, axis/road, general area), facility type (hospital, health center, health post, mobile clinic), location, date, and category of attack (pillage, threat, assault, murder, arrest/detention, theft, blockade, or infrastructure damage). We recorded multiple categories for attacks where applicable.

We generated descriptive statistics on the number, type, and characteristics of attacks in order to summarize patterns in the nature and distribution of violence against healthcare across time and geographic areas. Additionally, key information of specific attacks and facilities are provided to aid interpretation of results and ensure findings are appropriately understood within the operational and security environment in which they occurred. Contextual information on attacks was drawn from the same sources from which we derived the attacks.

#### Health facility classification

We classified facilities into two categories: hospitals and referral-level facilities, and primary-level facilities. Hospitals and referral-level facilities include three hospitals (one per prefecture) and two referral health centers (in Sikikide and Tiringoulou) in Vakaga. Primary-level facilities included health posts, mobile medical units (MMUs), and non-referral health centers, as they provided more similar packages of services. Two MMUs, PK3 in Haute-Kotto and MMU PK8 in Ouaka, operated 24/7 with emergency staff and additional IMC personnel during the day.

### Analytical approaches

We applied four analytical approaches to assess the impact of attacks on healthcare services: (1) Visual analysis of service utilization trends before and after each attack, (2) Calculation of immediate change in utilization following an attack, (3) Interrupted Time Series analysis of service utilization before and after each attack, and (4) Survival analysis to examine time to the first reported case of an epidemic prone disease, in this case, measles. For the first three methods, we focused on key primary healthcare services for which we had sufficient DHIS2 data: outpatient consultations, ANC1 visits, facility-based deliveries, and MCV1 vaccinations. For hospitals and referral-level facilities, we also included in-patient hospitalizations as an outcome of interest. For the survival analysis, a nationwide measles outbreak occurred over the study period and the indicator assessed was time to first reported measles case. Table 1 provides an overview of the number and type of facilities assessed for each analytical approach.

**Table 1:** Facility inclusion by analytical approach and Prefecture

Approach	Facility Level	Haute-Kotto	Vakaga	Ouaka
Visual Analysis of Utilization Trends	Hospital/ Referral	1	3	1
	Primary/ MMU	17	18	8
Immediate Change Post-Attack	Hospital/ Referral	1	3	-
	Primary/ MMU	11	7	2
Interrupted Time Series Analysis	Hospital/ Referral	-	1	-
	Primary/ MMU	6	-	1
Survival Analysis	Hospital/ Referral	-	3	-
	Primary/ MMU	-	17	-

*All facilities were supported by International Medical Corps continuously or imntermittently from 2016 to 2020.*

*MMU – Mobile Medical Unit*

#### Visual analysis of service utilization trends before and after each attack

We conducted a visual analysis by graphing monthly service utilization for each facility before and after each attack. This approach provided a straightforward and accessible way to assess changes in utilization over time, including the direction and magnitude of change for specific services within individual facilities. These graphs allowed us to examine trends that could not be meaningfully summarized by a single metric. Visualization allowed us to assess service-specific and facility-level impacts over time, highlighting variations in disruption and recovery across settings.

Results are presented for three attacks that capture types of impact observed across facilities.

#### Calculation of immediate change in utilization following an attack

To quantify immediate change in key indicators after each attack, we calculated percent change by comparing data from the month prior to attack to the month after. For example, if an attack happened in March 2020, we calculated proportion change in indicators from February to April 2020.

Multiple attacks in consecutive months on the same facility were considered as a single instance with proportion change calculated from the month before the first attack to the month after the last. For example, if attacks happened in March and April 2020 on the same facility, the proportion change was calculated from February to May 2020.

Analysis was restricted to attacks that occurred in the facility or involved on-duty personnel, had a known month and year of attack, and had facility data available for the required time period. Attacks reported in the first and last months of study period (January 2016 and December 2020) were excluded due to the lack of pre- and post-attack data. Where pre-attack values were zero due to likely missing data (e.g., report transmission failures or record loss during pillage), we substituted the previous month’s values based on organizational documentation. Of 127 attacks identified, the final analysis covered nine attacks on hospitals or referral-level facilities, and 31 attacks for primary-level facilities.

#### Interrupted time series analysis of service utilization

To estimate long-term effects of attacks, we used an interrupted time series (ITS) approach using data before the attack to compare the expected and observed values in the 12 months following an attack. For each attack and indicator of interest, we fit a generalized additive model, assuming AR1 correlation structure and negative binomial distribution. The model included terms for monthly trend, and both immediate level change and monthly trend change following an attack. Seasonality was modeled using four cyclic cubic regression splines. The model was fit using mgcv package in R [31].

To estimate the difference between observed values and counterfactual (expected values had the attack not happened), we generated 1000 draws using a parametric bootstrap procedure [32] from a model setting the two attack coefficients to 0. For the 12 months following an attack, we generated the median and 95% prediction intervals for both the absolute and proportional differences between observed and counterfactual values. The proportional difference was calculated as the absolute difference between the observed and counterfactual values, divided by the counterfactual values.

Where possible, we compared pre- to post-attack change with changes in a control facility, defined as a facility providing similar services in a similar geographical location with an expected shared secular (non-attack related) trend. For referral-level facilities, we considered the nearest referral-level facility in the same prefecture as the one attacked. For a primary level facility, we considered facilities on the same peripheral axis (major road/direction), as different armed groups controlled different axes during the period. Facilities that experienced an attack in the period before or within twelve months after the attack of interest were excluded as controls.

Models were fit independently for attacked and control facilities, with the interruption date set to date of attack. To allow for comparison, we included an offset in each model equal to the facility’s mean number of consultations during the pre-attack. Fitted models and counterfactuals are presented graphically to allow visual comparison of trends.

Due to strict exclusion criteria—requiring at least 12 months of data before and after the attack and no other attacks in that period—only a small subset of facilities and attacks were included; to our knowledge, these facilities had no missing data as post-attack zeros were verified through organizational reports as true service suspensions rather than missing values.

#### Survival analysis of first reported case of an epidemic prone disease (measles)

When the health system is disrupted by attacks on healthcare, the risk of vaccine-preventable and epidemic-prone diseases increases, potentially contributing to avertible mortality. Between February 2019 and January 2020, CAR reported 3,653 measles cases, with an outbreak declared in 2019 in five districts [33]. The measles outbreak provided an opportunity to analyze the time from attack to the first facility-reported measles case. We analyzed data at health facility level, defining exposure as any attack on healthcare in 2018 (the year prior to the outbreak). There were no measles cases reported at an IMC facility in 2020, likely due to gaps in facility surveillance or reporting data, since measles cases in Vakaga were reported elsewhere [34]. Thus, analysis was restricted to facilities with at least six months of reporting or at least one measles case in 2019. We excluded facilities not supported by IMC for pediatric health services during the study period. Vakaga prefecture was the only area with data to support survival analysis.

To distinguish true zeros from missing data, we used weekly outpatient consultations—reported on the same forms—as a proxy for facility reporting, treating weeks with zero consultations as non-reporting and excluding facilities with more than half of weeks missing. Missing or non-reporting was relatively limited in the remaining facilities, as shown in Figure S1; given that gaps were often reported retroactively and that measles is a notifiable disease, we considered it unlikely that suspected or confirmed cases would go unreported. Thus we treated the first reported case as the true first case, supported by consistency with internal monthly reports.

We conducted survival analysis comparing time to first reported measles case between exposed and unexposed facilities. We hypothesized that facilities with an attack had lower MCV coverage, leading to an increased number of susceptible children and earlier measles introduction. Statistical testing and survival curve plots were generated using survival [35] and ggsurvfit [36] R packages, respectively.

## Results

### Profile of identified attacks

#### Number and source of attacks

We identified many more attacks than reported in existing databases, with 127 attacks across five years in the three prefectures (Table 2). The majority of attacks were identified from IMC internal reports and communications (72 attacks, 57%), followed by key informant interviews (47 attacks, 37%) (Table 2). Most attacks were reported by a single source (110 attacks, 87%), while 13% were reported in two data sources. Only five relevant attacks (4%) appeared in Radio Ndeke Luka.

**Table 2:** Source of attack data. Overall number corresponds to de-duplicated number of attacks identified

Source of data on attacks	Overall, *N* = 127^1^
IMC internal reports and communications	72 (57%)
Key Informant Interviews	47 (37%)
Insecurity Insight	13 (10%)
Anonymous source	6 (5%)
Radio Ndeke Luka	5 (4%)

^1^n (%)

#### Time and place of attacks

**Fig. 2:**
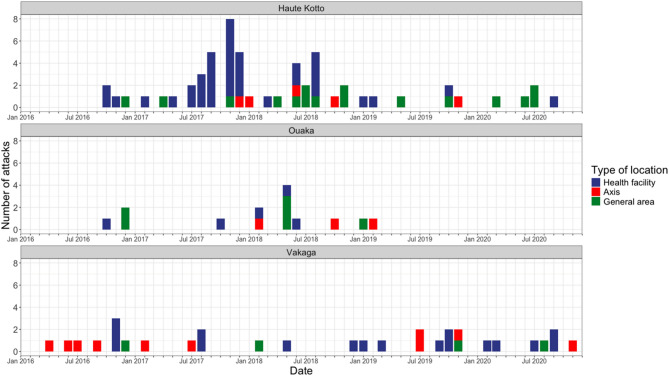
Identified attacks on healthcare in catchment areas and peripheral axes supported by International Medical Corps, from 2016 to 2020, by prefecture and by year

Figure 2 presents the number and type of reported attacks on healthcare by month from January 2016 to December 2020 across the three prefectures. The highest concentration of attacks was observed in Haute-Kotto, particularly between mid-2017 and mid-2018, where most attacks involved health facilities. In contrast, attacks in Ouaka and Vakaga were fewer and more sporadic. The timing and distribution of attacks appeared to reflect broader conflict dynamics: most attacks in Haute-Kotto occurred in 2017–2018, while in Vakaga, attacks were more frequent in 2019–2020. Across all prefectures, 61% of identified attacks took place at a health facility or involved on-duty personnel, with the remainder occurring along peripheral axes (17%) or in general areas (22%). Attacks on axes primarily involved mobile medical units (MMUs) traveling on roads, while general area attacks included attacks such as the looting of IMC bases and obstruction of IMC teams or patients by armed groups.

#### Type of attack

Some facilities experienced multiple attacks during the study period. For example, in one hospital, we identified ten separate attacks, including occupation, threats, physical aggression, gunfire, and clashes. Of the 127 attacks, 36 (28.3%) fell into multiple categories. Across the categories of attack, removal or attempted removal of assets the most common (26.7%), followed by threats (18.2%) and pillage (16.5%) (Table 3). Physical or sexual violence—including aggression, murder, and arrest, detention, torture, or kidnapping—occurred in 10.2%, 7.2%, and 5.7% of attacks, respectively. In total, some form of physical or sexual violence was reported in 23.3% of attacks, with 13 instances of murder.

**Table 3:** Identified attacks on healthcare, by attack category and health prefecture*

Attack category	Overall, *N* = 176^1^	Haute-Kotto, *N* = 75^1^	Ouaka, *N* = 42^1^	Vakaga, *N* = 59^1^
Removal or attempted removal of assets	47 (26.7)	20 (26.7)	9 (21.4)	18 (30.5)
Threat	32 (18.2)	19 (25.3)	2 (4.8)	11 (18.6)
Pillage	29 (16.5)	9 (12.0)	8 (19.0)	12 (20.3)
Physical or sexual aggression	18 (10.2)	5 (6.7)	7 (16.7)	6 (10.1)
Murder	13 (7.2)	5 (6.7)	6 (14.2)	2 (3.4)
Arrest, detention, torture, or kidnapping	10 (5.7)	4 (5.3)	1 (2.4)	5 (8.5)
Intrusion, occupation or clashes	9 (5.1)	4 (5.3)	5 (12)	0 (0)
Road blockage of ambulance / Extortion	8 (4.5)	5 (6.7)	1 (2.4)	2 (3.4)
Bombing, gunfire or arson	5 (2.8)	3 (4.0)	2 (4.8)	0 (0)
Damages	5 (2.8)	1 (1.3)	1 (2.4)	3 (5.1)

*A single instance of an attack (*n* = 127) could be classified under multiple categories of attack. “Removal or attempted removal of assets” category includes theft or confiscation of goods, robbery or attempted robbery, and burglary or attempted burglary

^1^n (%)

### Visual analysis of service utilization trends pre- and post attack

Changes in utilization varied greatly across services, facility-level, and time. We observed three general patterns of service utilitzation following an attack: complete and prolonged facility shutdowns (e.g., Ngoubi Health Post), long-term disruptions in specific services (e.g. Irabanda Health Post loss of routine vaccination services), or no disruptions or short-term disruptions (e.g., Madomale Health Post). There were many variations and differences in the degree of severity within these patterns, reflecting the diverse ways attacks impacted service delivery across facilities.

**Fig. 3:**
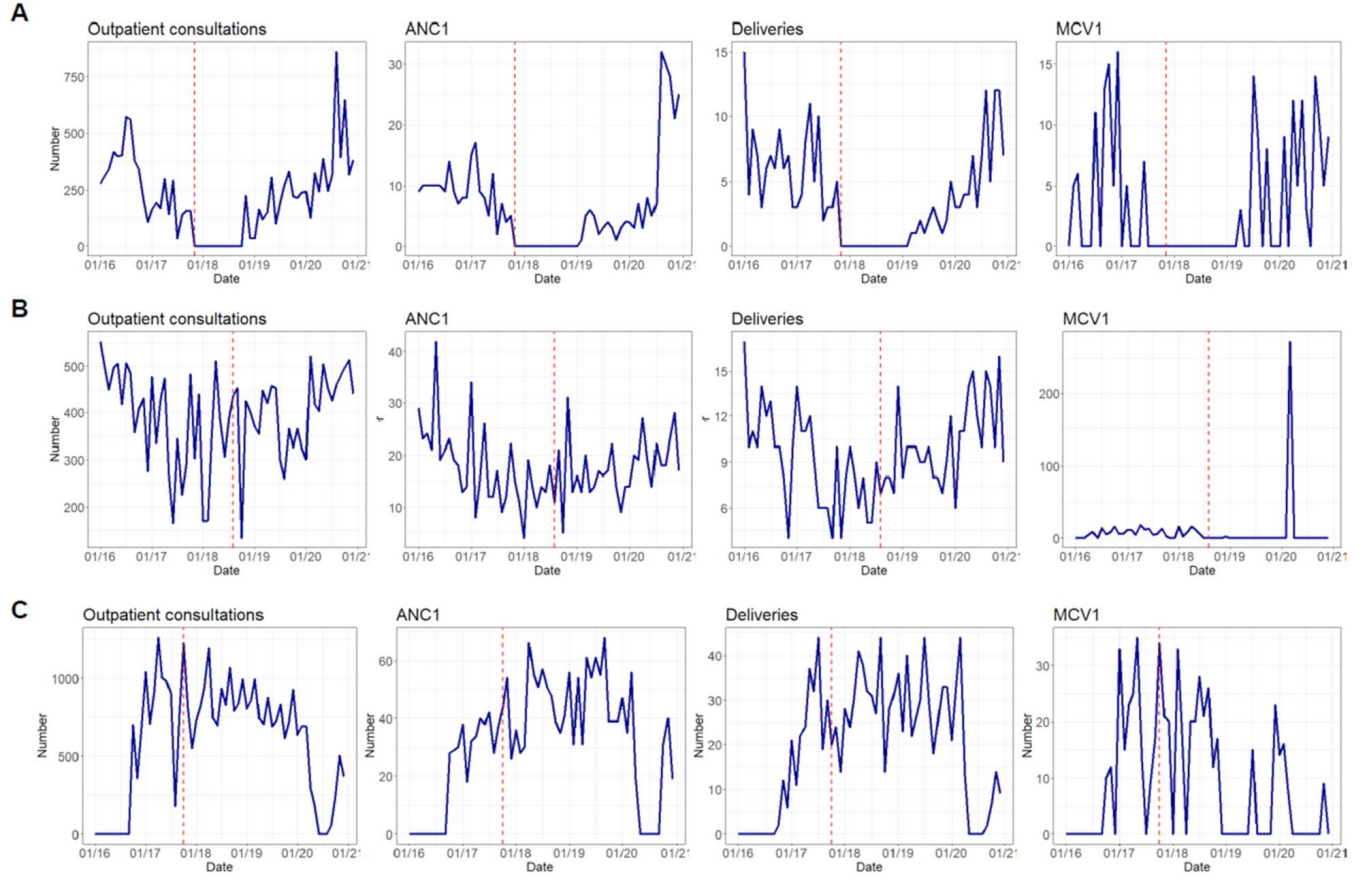
Illustrative examples of service utilization change in three health posts in Haute-Kotto and Ouaka prefectures following an attack on healthcare. **(A)** Complete shutdown of services. Panel shows monthly trend for outpatient consultations, first antenatal consultation (ANC1) visit, deliveries, and first dose of measles-containing vaccine (MCV1) in Ngoubi Health Post (Haute-Kotto), pillaged November 2017. **(B)** Long-term disruption to specific services. Panel shows monthly trends for health services in Irabanda Health Post (Haute-Kotto), pillaged August 2018. A mass vaccination campaign took place in April 2020. **(C)** No observable or very short duration of change in service utilization. Panel shows monthly trends for health services in Madomale Health Post (Ouaka), pillaged October 2017. * Red dotted line represents month of attack

In Ngoubi Health Post, an armed group pillaged the facility in November 2017 causing all health personnel to flee and halting services for over a year. The visual mapping of service data from Ngoubi Health Post (Haute-Kotto) shows cessation of services, outpatient consultations, ANC1, deliveries, and MCV1 vaccination (Fig. 3A). In August 2018, Irabanda Health Post experienced multiple attacks during which the facility head was assassinated in an ambush and the facility was looted. The mapping illustrates no discernible change in utilization of outpatient, ANC1, or delivery services following the cluster of attacks. However, MCV1 utilization dropped to zero and remained suspended, with the exception of a vaccination campaign in early 2020 (Fig. 3B). The termination of vaccination services was due to gaps in the cold chain, as the health district and IMC teams were unable to deliver vaccines due to revised security protocols. In contrast, for Madomale Health Post, there is no observable change in the pattern of service utilization before and after the attack that occurred in October 2017 (Fig. 3C). Instead, fluctuations in utilization of services are similar in the pre-and post-attack periods.

### Estimating immediate change in service utilization following an attack on healthcare

**Fig. 4:**
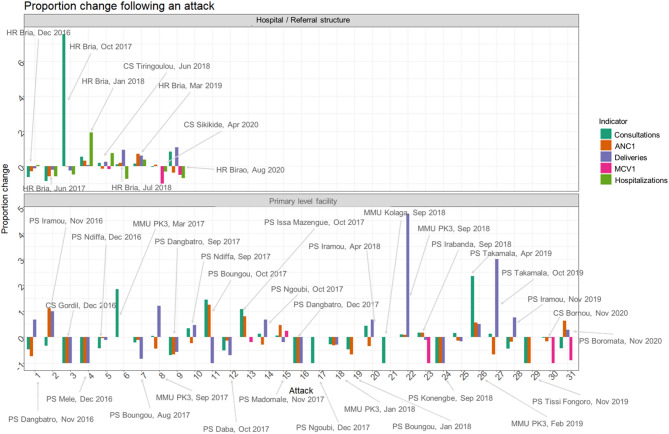
Immediate change in services following an attack on healthcare, by health service. Immediate change calculated as proportion difference in indicator in the month before and after attack. Labels indicate facility associated with the attack and the month of attack. For instances where multiple attacks occurred over consecutive months, the date indicates month of first attack

As with the changes over time, results for immediate impacts varied across services, facility level, and time (Fig. 4). Proportional change ranged from − 100% (full stop of services) to a 655% increase in pre-attack levels. For hospitals and referral-level facilities, we observed no decreases > 25% in deliveries, while in two of three attacks, MCV1 levels decreased by 50% and 100%. Changes in hospitalizations, outpatient consultations, and ANC1 were mixed.

For primary-level facilities, MCV1 decreased by more than 25% for three out of five attacks. Changes in consultations, ANC1, and deliveries showed no clear pattern. For many attacks, indicators changed in the same direction and magnitude (for example, 50% increase or decrease in deliveries, ANC1, and consultations), but this was not always the case.

These estimates of immediate changes should, however, not be interpreted as direct impacts of attacks, as other factors may explain the observed differences. For example, the 655% increase in outpatient consultations was observed at HR Bria following multiple attacks in July through September 2017. During this period, there was widespread insecurity, displacement, and clashes across the area, while a ceasefire agreement in September 2017, accompanied by peacebuilding activities, may have increased healthcare seeking and access in October 2017. Furthermore, the “pre-attack” month, May 2017, was characterized by intense violence, including large-scale displacement of population, suggesting the observed 655% increase reflects broader secular trends rather than effects of the attack alone.

### Interrupted time series analysis of service utilization

We analyzed eight facilities with at least 12 months of pre- and post-attack data, including six in Haute-Kotto, one in Ouaka, and one in Vakaga. One was a referral-level facility, and seven were primary-level. Further details on facility selection and model fits are presented in the supplement (Appendix 2).

**Fig. 5:**
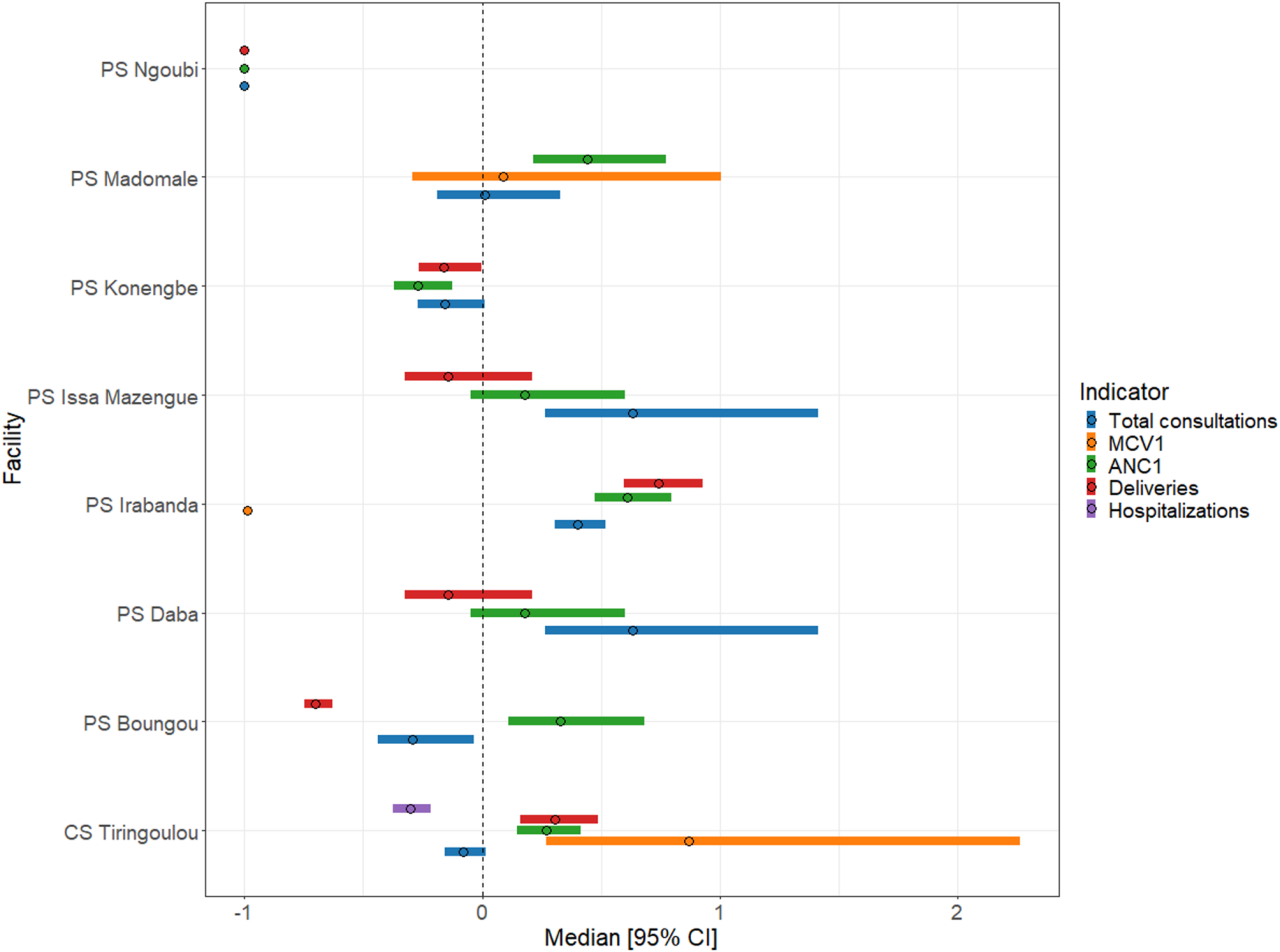
Forest plot presenting median and 95% confidence intervals for proportion difference in observed and expected (counterfactual) outcomes in 12 months following an attack on healthcare. The black point represents the median, and the bar indicates the span of the confidence intervals

In four facilities (Madomale, Konengbe, Boungou, and Tiringoulou), the median observed post-attack consultations were lower than the counterfactual, with Ngoubi and Boungou Health Posts experiencing > 25% reduction (Fig. 5). In contrast, three facilities (Issa Mazengue, Irabanda, and Daba) experienced a > 25% increase compared to the counterfactual. One facility, Ngoubi Health Post, was completely shut down for the year, with a 100% reduction in consultations, ANC1, and deliveries. Overall, there was no consistent direction of change across indicators.

**Fig. 6:**
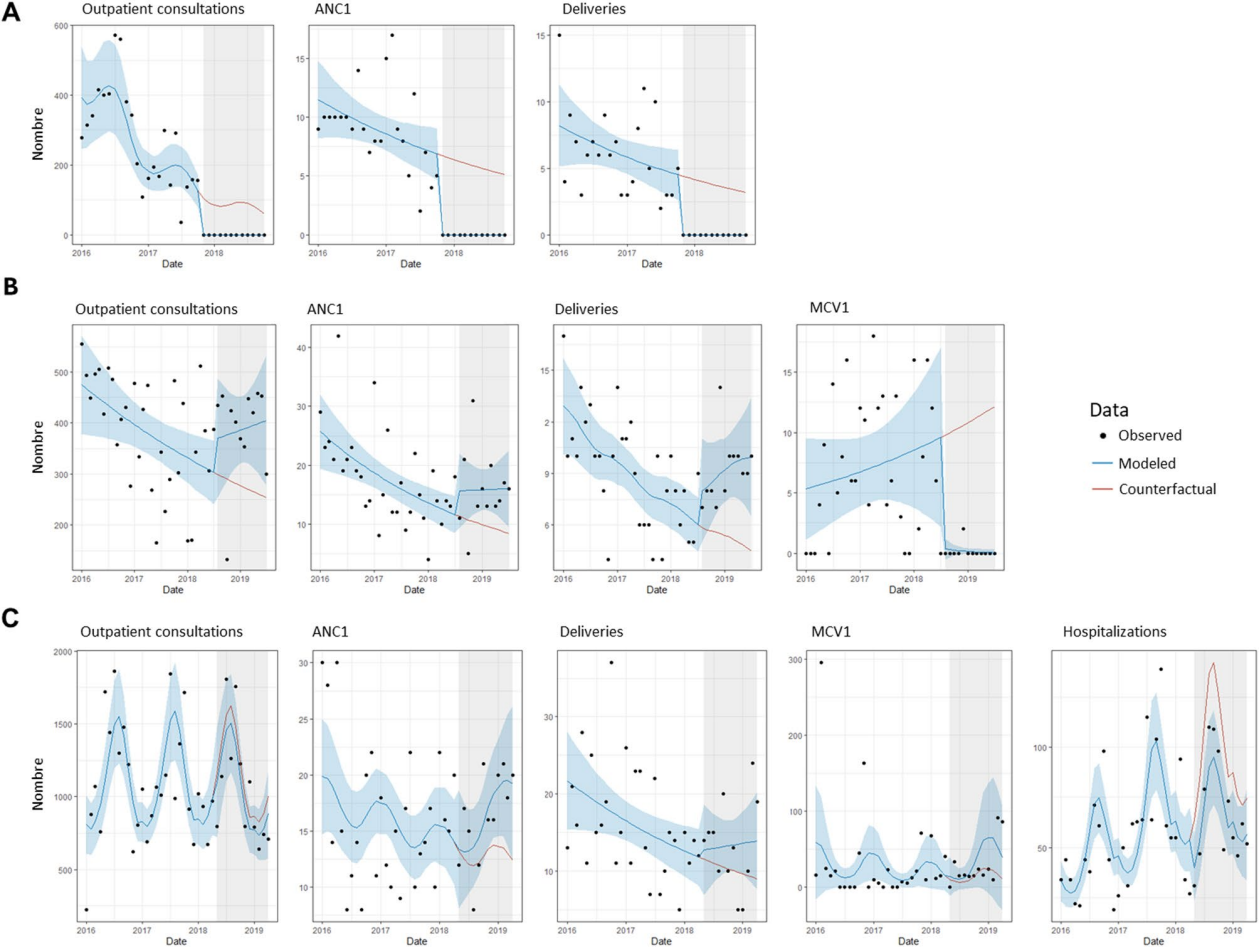
Interrupted time series for three health facilities that experienced attacks between 2016 and 2020. Panels display observed values for consultations, ANC1, deliveries, MCV1, and hospitalizations. Blue line and ribbon indicate median and confidence intervals of the fitted model; red line is the median counterfactual value for the post-attack period. **A**. Ngoubi Health Post, pillaged in November 2017. **B**. Irabanda Health Post, which experienced pillage, robbery, and murder in August 2018. **C**. Tiringoulou Health Center (referral-level facility), pillaged in May 2018

We considered three specific examples to illustrate results. The attack on Ngoubi Health Post in November 2017 is again demonstrated in Fig. 6A. The attack was associated with an estimated loss of 1000 + consultations, with a median loss of 45 deliveries and 71 ANC1 visits. Key challenges for restarting services were loss of materials and supplies, population displacement, and health personnel fear of resuming until the population returned. In late 2018, MSP and IMC carried out exploratory visits, outreach activities, and efforts to encourage personnel to return. Activities restarted in February 2019.

In August 2018, despite the attacks on Irabanda Health Post, which included assassination and pillage, we did not observe a decrease in consultations, ANC1, or deliveries, although as described in the visual analysis, MCV1 vaccinations ceased due to disruptions in the cold chain (Fig. 6B).

Tiringoulou Health Center, a referral facility, was raided in May 2018, during which perpetrators neutralized three personnel and pillaged the facility. Despite this, the damage was limited to the removal of medications and supplies such as mattresses, with no structural destruction. Its proximity to an airstrip allowed for a stable supply chain and rapid recovery, and post-attack data showed only minor decreases in consultations and hospitalizations, along with slight increases in ANC1, assisted deliveries, and MCV1 vaccinations (Fig. 6C).

**Fig. 7:**
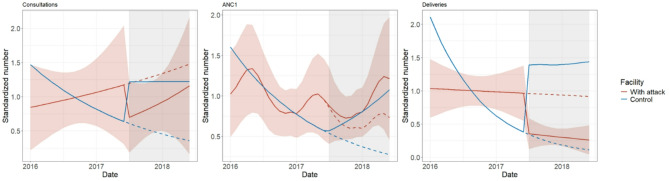
Interrupted series for Boungou Health Post, which experienced a murder of health facility personnel in July 2017, and control facility in Haute-Kotto prefecture. Control facility did not experience an attack during this time period. Solid line represents the model fitted to observed data during the post-attack period, while dashed line is the fitted counterfactual

The analysis was limited by the difficulty in finding control facilities, especially in Haute-Kotto, where most facilities experienced attacks in 2017–2018. For Boungou Health Post, where a health worker was murdered in July 2017, we identified one health center to serve as a plausible control. While Boungou saw declines in both outpatient consultations and deliveries in the twelve months post- attack, the same drop was not observed in the control facility (Fig. 7).

### Comparison of time to first measles case for facilities that did and did not experience an attack, Vakaga prefecture

**Fig. 8:**
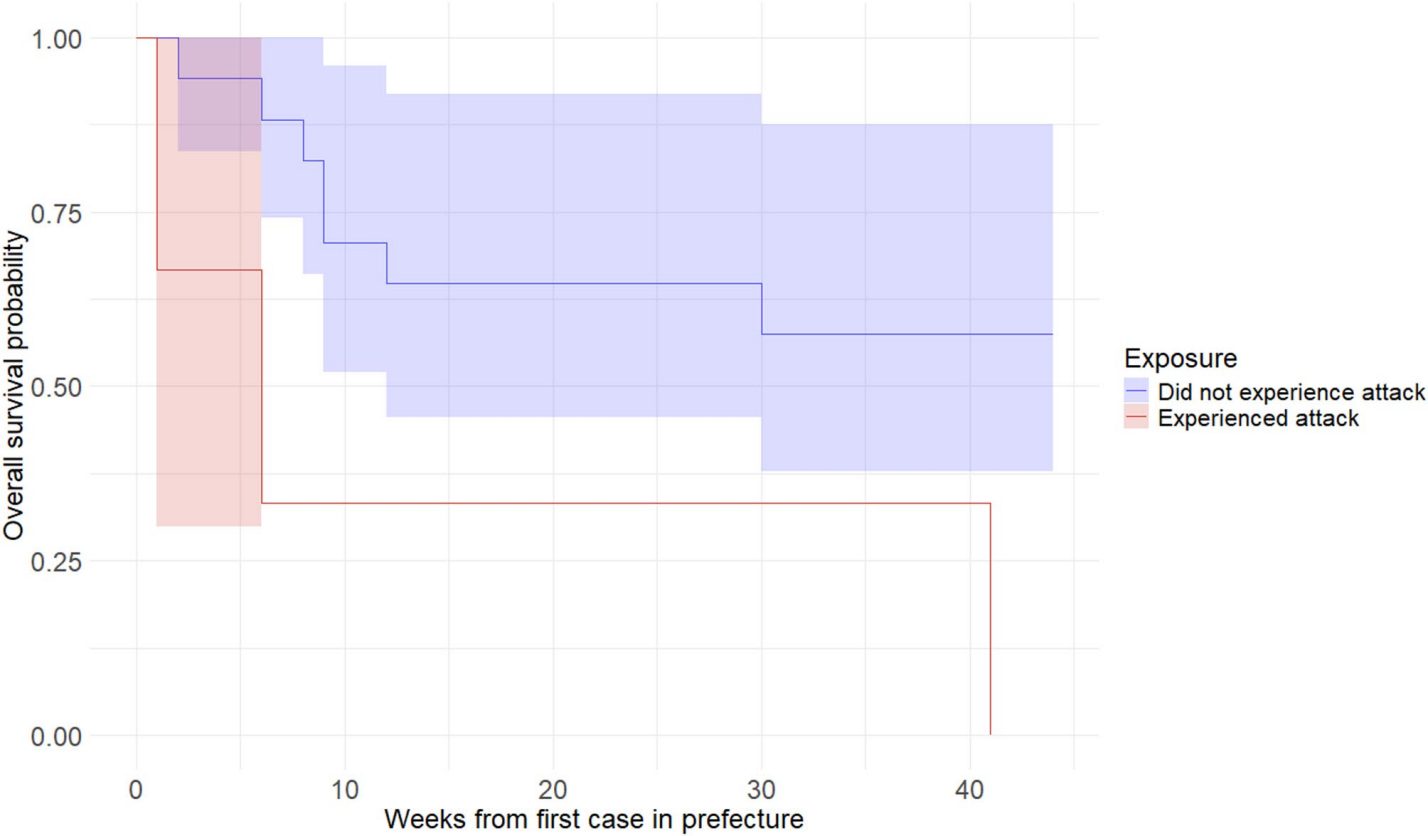
Survival curves for health facilities that did and did not experience attacks, Vakaga prefecture. Outcome is defined as reported measles case; exposure is defined as a reported attack on healthcare in 2018. Origin is time of reporting of first measles case in the prefecture

Among twenty facilities analyzed, nine reported at least one measles case during the 2019 outbreak. A swimmer plot depicting time to first measles case is presented in the Supplement (Figure S1). The log-rank test for difference in survival curves suggests a difference in time to first measles case (*p* < 0.001) (Fig. 8); however, results should be interpreted with caution due to the small number of exposed facilities (3 attacked in 2018), compared to unexposed (17 without attacks).

## Discussion

### Main findings

#### Variable impacts of attacks on healthcare

The frequency and distribution of attacks aligned with periods of heightened conflict in the prefectures, peaking in Haute-Kotto between 2017 and 2018, in Vakaga in 2016 and 2019–2020, and in Ouaka in 2018 and 2020. The patterns reflect how local conflict dynamics influence the targeting of healthcare, and may be shaped by the presence of armed groups, shifting frontlines, and political control of the territory. Regional differences in healthcare quality, population characteristics, and context alongside variations in attack type, intensity, and recurrence influence the degree of disruption observed across sites.

Some facilities experienced multiple attacks during the study period, further complicating recovery and continuity of services. One hospital was subject to ten separate incidents, including occupation, threats, physical aggression, gunfire, and armed clashes. These repeated assaults likely compounded their operational impact, even if individual attacks were not always immediately disabling. Across all 127 attacks, 36 (28.3%) fell into multiple categories, underscoring the complexity and layered nature of violence faced by health facilities. These cumulative and complex patterns are difficult to quantify using routine health data alone. Additional qualitative research may help to explain why certain attacks result in greater disruption than others and how factors such as remoteness, supply chains, or community support influence health system response and recovery.

While the most common types of attacks did not involve physical or sexual violence, a substantional portion, 23.3%, did. Notably, we identified 13 instances of murder—an alarming finding given that these deaths may not have been captured by existing surveillance systems. Rates of personal violence were higher than those documented in Ukraine during the first 18 months of the conflict, with 7.2% of attacks in our study involving murder (compared to 2% in Ukraine) and 15.9% involving other forms of aggression (compared to 4% resulting in injury in Ukraine) [14].

Our findings suggest that in CAR there were variable effects in how attacks affected healthcare. Some attacks led to complete facility shutdowns, others affected specific services, and some had minimal or short-term impact. The variation in impact is illustrated by cases like Tiringoulou Health Center, which experienced minimal disruption to services due to mitigating factors such as proximity to an airstrip, which ensured a stable supply chain. In contrast, insecurity and poor access likely delayed resumption of services in more remote areas.

Service level differences were also observed. Facility deliveries were less affected likely because births cannot be postponed, delivery care is less reliant on supply chains, provider skills retain value even with limited resources, and maternity units are often staffed by trained community members. These findings are consistent with research in Syria [20] and Uganda [12] which found assisted deliveries may be less disrupted by violence than other health services and may even increase during periods of insecurity.

In contrast, immunization services were particularly vulnerable, experiencing significant declines due to supply chain disruptions or loss of personnel, followed by surges once services resumed. Attacks often led to stricter security protocols for health actors, including restricted road movements, which prevented delivery of vaccines and supplies.

#### Building resilient health systems

Although this study did not assess the effectiveness of mitigation measures, the findings suggest that rigid, one-size-fits-all approaches are unlikely to work. Responses should be flexible and adaptive. Given that pillaging is common, strengthening supply chains is critical to safeguard vaccination programs and other services. This is especially urgent in CAR, where routine vaccination coverage remains precarious. In 2024, national measles vaccination coverage was estimated at 25.5% [38]. A 2021 assessment in Vakaga found that 13 of 21 facilities lacked functional vaccine storage, with stockouts exacerbated by reliance on nearby facilities or mobile clinics. Insecurity also disrupts movement of supplies, making vaccination coverage difficult even in the absence of direct attacks [13].

Tailored mitigation plans can help reduce the long-term impacts of attacks on health services. Findings from the in-depth interviews emphasize the role of local communities and healthcare workers in maintaining services after attacks [6]. Humanitarian organizations and donors should prioritize flexible funding and capacity-building initiatives to strengthen local resilience. Despite experiencing consecutive attacks, including murder and pillage, Irabanda Health Post remained fully operational, with the exception of vaccination, due to the dedication of its staff and community. Prior to the attack, the facility was supported by an Integrated Community Case Management project that emphasized local capacity building. Additionally, its remote location left residents with few alternatives, potentially motivating staff to continue operations despite security risks. Engaging local actors in mitigation and response efforts will improve their success.

Effective responses should consider the type of attack and the services affected. Strengthening vaccine supply chains could help maintain immunization rates, while enhancing emergency obstetric care will better address disruptions to maternal services. Other approaches to strengthening health system resilience may include investment in cold chain infrastructure for each facility to reduce dependency on external supplies, expansion of community health initiatives, and integration of a minimum service package for vaccination in remote or insecure areas. Mobile clinics can serve as temporary solutions when services are disrupted. In Irabanda, we observed a spike in vaccinations more than a year after suspension of services due to mobile clinic support. Finally, advocacy, mediation, and dialogue with armed groups are critical to protecting of personnel, patients, and caregivers, and securing access to care within facilities and for outreach services [39].

### Data collection and triangulation of attacks on healthcare

Gathering data on attacks in conflict zones is challenging, often leading to incomplete and inconsistent reporting. We combined primary and secondary sources, including IMC internal reports, Insecurity Insight documents, Radio Ndeke Luka publications, and an anonymous database, to create a more comprehensive dataset. Research in Syria similarly showed that combining data sources improved attack documentation [40]. We triangulated sources, conducted multiple interviews per attack, and systematically extracted variables across sources to improve data accuracy, completeness, and reliability.

Access to quality facility-level health data is a precondition for quantitative analysis of service utilization. We used weekly aggregated data from IMC’s health information system (HIS). Despite limited resources, we documented 127 attacks across just three prefectures from 2016 to 2020, compared to 80 attacks recorded by WHO across the entire country during the same time period [37]. Only 13% of attacks matched across multiple sources, highlighting the importance of using multiple sources and different methods of data collection to obtain a comprehensive picture of the landscape of attacks. Most attacks in this study were identified only in IMC attack data, which like other service provider records is rarely shared externally.

Some international NGOs have made efforts to improve data transparency. However, the lack of systematic data-sharing indicates major structural problems with reporting, leading to severe underestimation of the scale of violence against healthcare. In addition, access to HIS data requires partnerships with local or international NGOs or district health teams. In conflict settings, HIS are often disrupted [41], and actors may be reluctant to share data [42], introducing biases based on geographic access or population characteristics.

Without accurate reporting, tracking attacks, allocating resources, and developing mitigation strategies remains difficult. Parada et al. compared two datasets of attacks on healthcare, derived from publicly-reported attacks on healthcare, and found only a 13% overlap, underscoring the underreporting of attacks and inconsistency in datasets resulting from variation in sources of data and curation processes [43]. One scoping review identified systematic data collection as a critical recommendation for response to attacks [44]. Our study reinforces this recommendation: while we identified 127 attacks in study areas, Insecurity Insight recorded no events in Vakaga and 32 events in Ouaka and Haute-Kotto prefectures in the same time period, most of which occurred outside catchment areas of IMC-supported facilities.

### Improving reporting mechanisms for attacks on healthcare

WHO’s *Surveillance System of Attacks on Healthcare* (SSA) was established to comprehensively document attacks, but has been rightly criticized for failing to achieve its objective [45, 46]. The number of attacks identified in our study greatly supercedes that reported by the SSA; specifically, the SSA reported 80 attacks from 2016 to 2020 across the entire country [37]. Recent research from Ukraine on attacks on healthcare, which relied solely on the WHO SSA, found lower levels of physical violence than our study [14]. This may be due to differences in the context, or it may be further evidence of the SSA’s shortcomings in comprehensively documenting attacks. Our findings underscore the need to assess and improve the SSA’s methodology, including greater outreach to and communication with international and local NGOs, civil society groups, and health providers. WHO must adopt a more transparent and cooperative approach data collection and reporting - one that does not compromise confidentiality or security but facilitates broader sharing and accountability [47].

Establishing a functional, reliable, and secure global mechanism for documenting attacks on healthcare is essential. Data sharing and transparency can be reinforced by formal agreements signed between NGOs, donors, and global health actors like WHO. Inadequate reporting creates a dangerous false perception that attacks are infrequent, minor, or lack significant long-term effects.

Governments, humanitarian actors, researchers, communities, and donors must collaborate to enhance data collection and share incident reports to fully understand the impact of these attacks. Triangulating data provided a more comprehensive picture of their scale and extent. Real-time monitoring, higher-frequency reporting, and zero-reporting guidelines can improve data accuracy and completeness, enabling more robust analysis. Integrating data on attacks into digital health information systems like DHIS2 [48] could support sustainable and continuous tracking, faster response, and informed decision-making. Strengthening global reporting mechanisms, including the SSA, is essential to understand the true cost of these attacks.

Despite the challenges of conducting research in fragile settings, the findings emphasize the critical need for robust, data-driven assessments to quantify the impact of attacks on healthcare. Expanding these efforts will help to fortify protective measures for healthcare systems in conflict zones and improve advocacy for affected populations.

Efforts to improve reporting mechanisms should be complemented by advocacy to prevent and mitigate the impacts of attacks and to hold perpetrators accountable. Advocacy against violence targeting healthcare should be a global priority. Stronger advocacy is essential to raise awareness, demand accountability, and ensure that the protection of healthcare is central to humanitarian response, policy-making, and global health security. When attacks are under-reported or ignored, it becomes difficult to mobilize action, as advocacy cannot succeed without evidence. Improved reporting and strong advocacy are complementary and must go hand in hand to reduce the frequency and impact of attacks. Without urgent and sustained efforts on both fronts, the normalization of violence against healthcare will continue to undermine health outcomes and violate fundamental human rights. Similarly, advocacy is essential to ensure that mitigation measures are put in place, including supports for health workers and communities who have experienced attacks.

### Novel application of existing methods

In addition to the more common approaches of visual analysis and calculation of immediate change, this study applied ITS and survival analysis to explore the impact of attacks on healthcare. While these methods are well-established in public health, and have been used to quantify the effects of violence on healthcare before [20], their application remains relatively novel and underutilized. ITS enabled us to measure post-attack changes in healthcare utilization over one year, comparing observed outcomes to counterfactual scenarios without attacks. Survival analysis linked healthcare disruptions to vaccine-preventable diseases like measles. This approach is particularly valuable in fragile contexts where service disruptions can lead to outbreaks, increased maternal and neonatal mortality, and higher incidence of malnutrition.

Using a mixed-methods approach provided a more comprehensive understanding of the impacts. Quantitative analysis objectively measured the severity and extent of disruptions, while qualitative data contextualized findings, identifying factors that continued to influence healthcare utilization after attacks occurred. This integration highlighted direct impacts and response strategies, capturing both statistical trends and human experiences. Applying established public health methods in this context demonstrated how attacks have the potential to affect distinct health services, reinforcing the need for targeted, data-driven approaches to healthcare protection, mitigation, and recovery. Understanding these impacts allows interventions to be tailored to the nature and scope of disruptions, improving response in conflict-affected settings.

### Importance of quantitative assessment of attacks on healthcare

This work describes the quantitative component of a mixed-methods study researching the impact of attacks on healthcare in CAR. Understanding these impacts is crucial to safeguarding health and human rights, reducing suffering, and preventing avertible deaths, particularly in fragile settings where healthcare access and quality are already severely constrained. Qualitative studies have historically dominated research of attacks on healthcare [2–8] due to challenges obtaining reliable facility data from conflict zones [49] and difficulties isolating the effects on healthcare from broader conflict dynamics. While qualitative research provides valuable insights, it fails to capture the full scope and variability of the impact of attacks on health systems, service utilization, and population well-being, especially long-term.

With few exceptions [9, 10], quantitative work on the impact of attacks against healthcare has focused on contexts outside of Sub-Saharan Africa. For example, in Syria, Burbach et al. found that healthcare attacks were significantly associated with declines in outpatient and trauma consultations and facility births [13]. Ekzayez et al. found that in Syria, violence was associated with slight declines in outpatient and ANC consultations, and increases in deliveries and C-sections [20]. However, Syria’s pre-conflict health system was more developed, making direct comparisons difficult. Neglected crises like that across CAR receive the least research attention, despite facing some of the greatest needs and widest evidence gaps. This knowledge and data gap has serious implications, as insufficient evidence prevents well-informed interventions. By quantifying immediate and longer-term effects of attacks in CAR, this study demonstrates how quantitative methods, especially in conjunction with qualitative data, can enhance understanding of the true and differential costs of healthcare attacks – particularly for the affected population – and strengthen the evidence base for better, targeted interventions, and improving the protection of healthcare in conflict.

### Limitations/challenges

Our findings must be interpreted with caution due to several key limitations. Observed impacts may be influenced by unmeasured factors including the pre-attack facility conditions, geographic location, rurality, accessibility, attack duration and frequency, staffing, funding, and local dynamics. We could not control for surrounding insecurity, displacement, or socioeconomic disruptions, limiting our ability to define causal relationships between attacks and healthcare outcomes.

We also encountered substantial methodological challenges. The frequency of attacks made it difficult to find control facilities for the ITS analysis. In Vakaga, the clustering of attacks in 2020 limited our ability to assess long-term impacts. ITS assumptions were likely violated as successive attacks disrupted data continuity. Incomplete and inconsistent data further limited the study. The absence of zero-reporting, infrequent reporting, and periods when IMC did not support some facilities likely resulted in underreporting of minor attacks, such as verbal threats. Missing data made it unclear whether zero cases reflected reality or a failure to report, though we used proxy indicators (e.g., consultation data) to complete gaps where possible. These inconsistencies likely underestimated the cumulative impact of smaller, frequent attacks and obscured the severity of service disruptions.

The quality of secondary health facility data used in this study also posed limitations. Routine health information systems in conflict-affected settings may suffer from inconsistencies in reporting, gaps in coverage, and limited verification, which may introduce reporting bias or affect reliability of service utilization trends over time. Missing data on date of attacks meant data had to be aggregated by month, masking short-term fluctuations in healthcare utilization and reducing the precision of ITS analysis. Furthermore, the inability to quantify attack severity or account for overlapping attacks made it difficult to isolate individual effects. Multiple attacks likely compounded disruptions, complicating the analysis. Key informant interviews may be subject to recall bias, particularly for events that occurred several years prior, and reporting may be influenced by the respondent’s role, experiences, or relationship to the facility.

We were unable to fully account for support from private providers, UN agencies, or NGOs across the prefectures due to the absence of complete data on their presence and coverage. Restricting the analysis to IMC-supported facilities may have biased the study as these facilities may differ from facilities that do not receive external support or from privately managed facilities. IMC-supported sites likely benefit from improved reporting systems and operational capacity and may be more or less likely to be targeted, depending on the context. As such, the generalizability of our findings to the broader health system may be limited.

The study did not assess quality of care. While patient visits were recorded, it is not known if qualified providers delivered appropriate treatment. This limitation is particularly relevant given the observed surge in service utilization following some attacks, which coincided with peaks in regional violence. These increases may reflect displaced populations seeking care at IMC-supported facilities due to attacks on other nearby healthcare sites not included in this study. Such surges could have affected the quality of care provided, a factor not assessed here but acknowledged by the study authors. Given these challenges our findings likely underestimate the full extent of the impact of attacks on healthcare.

Additionally, the absence of strong quantitative signals of service disruption should not be interpreted as an absence of negative impact. These effects may be better captured through the complementary qualitative component of the study, which explored patient and provider perspectives on the consequences of attacks.

Finally, the study was unable to include population-level measures, which would have strengthened the analysis, due to the inherent challenges of collecting such data in conflict-affected settings like northern CAR. As a result, the findings may not fully capture the broader population-level burden of the impacts of the attacks on healthcare.

## Conclusions

Reducing the impact of attacks on healthcare requires stronger data utilization and political engagement. Establishing a functional, reliable, and secure global mechanism for documenting attacks on healthcare is essential. Strengthening real-time monitoring, integration into digital health systems, and data sharing will improve response and advocacy efforts. Humanitarian actors, governments, researchers, and donors must collaborate to ensure a more accurate understanding of the scale and impact of attacks. Additionally, responses must be tailored to the type of attack and affected services. Mitigation strategies and response efforts should have significant involvement from affected communities, as they are the first and primary responders to attacks. Advocacy, mediation, and dialogue with armed groups are also critical to securing access and protection for healthcare workers, patients, and mobile teams.

Addressing the crisis of violence against healthcare requires a firm commitment from all actors to respect the sanctity of life and uphold IHL. We call for urgent global collaboration and action to protect healthcare services in conflict zones and safeguard the lives of vulnerable populations. These findings highlight the critical need for robust, data-driven assessments of attacks on healthcare in conflict zones and neglected crises.

## Electronic supplementary material

Below is the link to the electronic supplementary material.

Supplementary Material 1

## Data Availability

Two sources of data for attacks on healthcare in CAR are publicly available: Insecurity Insight (https://data.humdata.org/dataset/car-violence-against-civilians-and-vital-civlian-facilities), and Radio Ndeke Luka (https://www.radiondekeluka.org/). Additional information on attacks, obtained from interviews and internal documents of International Medical Corps, is not available due to sensitive nature. Aggregate data on attacks are provided in the text of this publication. Aggregate health facility data as well as deidentified information on attacks may be available from the corresponding author on reasonable request.

## Abbreviations

ANC1: First Antenatal Care Visit
CAR: Central African Republic
HIS: Health information system
ICASEES: Institut Centrafricain des Statistiques et des Etudes Economiques et Sociales
IMC: International Medical Corps
ITS: Interrupted Time Series
MCV1: First dose of measles containing vaccine
MMU: Mobile Medical Unit
MSP: Ministry of Health and Population
NGO: Non-Governmental Organization
WHO: World Health Organization
