# Supplementary material for: Healthcare under fire: quantifying the impact of violence on medical services in facilities supported by International Medical Corps in three prefectures of Central African Republic, 2016–2020
Source: Confl Health. 2025 Aug 5;19:57. doi: 10.1186/s13031-025-00686-w (PMC12323227; doi:10.1186/s13031-025-00686-w)

# Supplementary materials

## Appendix 1. French version of article

Les soins de santé sous attaque: quantifier l’impact de la violence sur les services médicaux dans les établissements sanitaires soutenus par International Medical Corps dans les trois préfectures de la République centrafricaine, 2016 - 2020

Natalya Kostandova^1,2+^, Jennifer OKeeffe^3+^ , Audrey Mahieu^4^ , Blaise Bienvenu Ali^5^ , Christian Mulamba^6,++^ , Pierre Somsé^7^ , Odilon Guesset Bingou IV^5^ , Sebastien Dackpa^5^ , Gerard Mbonimpa^6^ , Thierry Fikiri^6^ , Larissa Fast^8*^, Leonard Rubenstein^2^

^1^ International Medical Corps, Washington, DC, États-Unis

^2^ Département d'épidémiologie, Johns Hopkins Bloomberg School of Public Health, Baltimore, MD, États-Unis

^3^ Département de la santé internationale, Johns Hopkins Bloomberg School of Public Health, Baltimore, MD, États-Unis

^4^ Centre d'études humanitaires de Genève, Université de Genève, Genève, Suisse

^5^ Institut centrafricain des statistiques et des études économiques et sociales, Bangui, République centrafricaine

^6^ International Medical Corps, Bangui, République centrafricaine

^7^ Ministère de la Santé et de la Population, Bangui, République centrafricaine

^8^ The University of Manchester Humanitarian and Conflict Response Institute, Manchester, UK

^+^ Les auteurs ont contribué à parts égales

^++^ Affiliation actuelle : International Medical Corps, Abuja, Nigeria

^*^ Auteur correspondant: [larissa.fast@manchester.ac.uk](mailto:larissa.fast@manchester.ac.uk)

## Résumé

**Introduction**

Les attaques contre les établissements de santé en République centrafricaine (RCA) sont généralisées, peu rapportés et insuffisamment traitées. Cette étude a examiné l'impact des attaques contre les établissements de santé dans trois préfectures touchées par le conflit – Ouaka, Haute-Kotto et Vakaga – entre 2016 et 2020, en évaluant les perturbations immédiates et à long terme.

**Méthodes**

Les perturbations dans les établissements de santé primaires et de référence ont été évaluées à l'aide des données du programme d'International Medical Corps (IMC) et des données sur les attaques de l'IMC, d'informateurs clés, de bases de données secondaires et des médias locaux. Les indicateurs clés – consultations externes, premières consultations prénatales (CPN1), accouchements en établissement, vaccination contre la rougeole (VAR1) et hospitalisations – ont été analysés à l'aide d'une analyse visuelle des tendances, d'une estimation de l'évolution immédiate et de séries chronologiques interrompues (SCI). L'analyse de survie a évalué l’association entre les attaques et le délai de déclaration du premier cas de rougeole lors d'une épidémie nationale.

**Résultats**

Au total, 127 attaques individuelles ont été identifiées sur cinq ans, principalement à partir des rapports de sécurité de l'IMC. Les incidents les plus courants étaient l’enlèvement ou la tentative d’enlèvement de biens (27 %), la menace (18 %) et le pillage (17 %). Au moins une forme de violence physique ou sexuelle a été documentée dans 23,2 % des attaques, avec 13 cas de meurtre. L'analyse visuelle a montré trois modèles d'impact : fermeture de l'établissement, perturbation de services spécifiques et changements minimes ou à court terme. Les changements immédiats variaient également, les changements de service allant de -100 % à 63 %. Dans l'analyse SCI, quatre des huit établissements ont affiché un déficit de plus de 25 % en consultations externes, tandis que trois ont affiché un excédent de plus de 25 %. L'analyse de survie a montré une différence significative dans le délai jusqu'au premier cas de rougeole entre les établissements attaqués et les établissements non attaqués (p < 0,001), bien que les résultats soient limités par la petite taille de l'échantillon. Dans l'ensemble, les services de santé maternelle ont connu moins de fluctuations, tandis que les services de vaccination ont complètement cessé dans certains établissements.

**Conclusion**

Cette étude démontre qu'il est possible d'améliorer l'identification et le profilage des attaques dans les contextes à faibles ressources et présente plusieurs approches pour quantifier leur impact. Elle met également en évidence les difficultés d'analyse, notamment les limitations importantes en termes de qualité et de disponibilité des données. Les résultats renforcent l'urgence d'une collecte systématique de données, d'une surveillance en temps réel, de stratégies d'atténuation adaptées au contexte et d'un soutien aux acteurs locaux qui maintiennent les services lorsque l'aide extérieure est limitée. Les recherches futures devraient s'appuyer sur ces résultats pour élaborer des stratégies de protection, d'atténuation et de rétablissement plus efficaces pour les systèmes de santé en zones de conflit.

**Mots clés**

Attaques contre les soins de santé, République centrafricaine, Conflits armés, Système de santé, études observationelles

## Introduction

Les attaques contre les services de santé dans les conflits armés constituent une menace critique pour la santé et le bien-être des populations vivant dans les pays touchés par un conflit. Ces attaques compromettent souvent la prestation des soins de santé et limitent l’accès aux services essentiels pour des populations déjà vulnérables^1^. L’ampleur et la durée des interruptions de services dans des contextes particuliers restent mal comprises. Une meilleure connaissance de ces effets pourrait contribuer aux efforts visant à atténuer les impacts de la violence et à mitiger ces effets.

Au cours de la dernière décennie, des études qualitatives ont évalué l’impact des attaques sur les personnels de santé, les services, les résultats et les capacités du système de santé^2-8^. Bien que ces études fournissent des informations précieuses, elles sont limitées dans l’évaluation des effets différentiels des attaques sur des services et des populations particuliers, ainsi que dans la durée des impacts. De nombreuses études ont porté sur les conséquences à court terme des attaques^15^ et sur les expériences des professionnels de santé^16-19^. Quelques études quantitatives ont cherché à mesurer l'impact à long terme des attaques^13, 14, 20^, la majorité d'entre elles ayant été menées au Moyen-Orient. Des études quantitatives menées en Afrique au Burkina Faso^9^, au Nigéria^11^ et en Ouganda^12^ se sont principalement concentrées sur l'impact des services de santé maternelle.

La rareté des études quantitatives sur les impacts de la violence contre les soins de santé est probablement due à la difficulté d’accéder à des données pertinentes sur la prestation et l’utilisation des services après les attaques en raison de l’insécurité, des barrières de communication et des politiques limitant le partage d’informations^1^. Dans de nombreux pays touchés par des conflits, en particulier en Afrique de l’Ouest et du Centre, les attaques contre les soins de santé, notamment les pillages, les menaces contre le personnel et l’accès restreint, sont si courantes qu’elles ne sont souvent pas signalées, ce qui entraîne des lacunes dans les données^1^. De nombreux pays ne disposent pas de systèmes de signalement fiables, et la peur des représailles dissuade encore plus les signalements. Lorsque des attaques sont signalées, la réponse et le soutien peuvent être minimes, voire inexistants^6^.

Cette étude comble directement plusieurs lacunes identifiées dans les travaux quantitatifs précédents. À quelques exceptions près^20^, l'analyse de série chronologique interrompue (SCI) ou l'analyse de survie ont rarement été appliquées pour examiner l'association entre les attaques et les résultats du délai avant l'événement, tels que l'apparition de maladies après les attaques. De même, les consultations externes ont rarement été analysées dans ce contexte. Les effets sur les services de santé non maternels, notamment le taux de vaccination, les hospitalisations et les cas de maladies à potentiel épidémique, ont rarement été quantifiés. En appliquant de multiples méthodes d'analyse et en examinant un large éventail d'indicateurs de services, cette étude apporte de nouvelles preuves dans un domaine peu étudié et offre une compréhension plus complète de la manière dont les attaques affectent la prestation de soins de santé dans les contextes fragiles et touchés par des conflits et de la manière dont ces effets peuvent être quantifiés. Peu d'études ont été menées sur les crises négligées et, à notre connaissance, aucune recherche n'a tenté de quantifier l'impact des attaques contre les soins de santé en République centrafricaine (RCA), un pays qui connaît une violence soutenue contre les soins de santé depuis des années^1, 21, 22^.

La RCA est l'un des pays les plus pauvres du monde, se classant au 191e rang sur 193 pays selon l'indice de développement humain des Nations unies^23^, avec le 5e taux de mortalité maternelle et infantile le plus élevé au monde^24^. Sur une population de 6,1 millions d'habitants, plus d'un million de personnes sont déplacées et plus de trois millions ont besoin d'une assistance humanitaire^25^. RCA compte 0,21 médecin pour 10 000 habitants, ce qui est nettement inférieur aux 25 médecins pour 10 000 habitants recommandés pour une couverture adéquate des soins de santé primaires^26^. Le pays est en proie à une guerre civile depuis plus de dix ans, caractérisée par une violence généralisée à l'égard des soins de santé, notamment des meurtres, des agressions physiques et sexuelles, des enlèvements, des incendies criminels, des bombardements, des pillages, des occupations et des menaces verbales^6^.

Le système de santé de la RCA est touchée par un manque de ressources et est fortement impacté par le conflit et l'instabilité en cours^24^. Il est structuré en trois niveaux – soins primaires, secondaires et tertiaires – mais l'accès reste limité, en particulier dans les zones rurales et touchées par le conflit^27^. La majorité des services de santé fonctionnels sont soutenus par des organisations internationales et non gouvernementales, avec des lacunes importantes en matière d'infrastructures, de personnel et de fournitures médicales. Le financement de la santé publique est faible et les systèmes d'information sanitaire sont défaillants, ce qui complique encore davantage la prestation et la planification des services. L'insécurité persistante a perturbé les services de routine et contribué à de mauvais résultats sanitaires dans tout le pays^27^.

L'étude s'est déroulée dans trois préfectures rurales - Ouaka, Haute-Kotto et Vakaga, sélectionnées pour leur présence significative de groupes armés, les déplacements importants de population et la prévalence des attaques contre les civils et les structures de santé^6^. Cette étude présente la composante quantitative d'une étude à méthodes mixtes examinant l'impact des attaques sur les soins de santé en RCA. L'étude analyse des attaques spécifiques et des données secondaires provenant d'établissements de santé soutenus par l'organisation non gouvernementale (ONG) International Medical Corps (IMC) afin de quantifier les effets à court et à long terme.

Grâce à des entretiens avec des informateurs clés, le volet qualitatif^6^ a révélé que les attaques étaient plus répandues que ce qui avait été rapporté, entraînant des fermetures prolongées d'établissements, des pertes d'approvisionnement importantes et des dommages disproportionnés pour les groupes vulnérables, notamment les enfants de moins de cinq ans, les personnes âgées, les personnes vivant avec un handicap, les personnes atteintes d'une maladie chronique et les personnes déplacées. Les travailleurs de la santé ont subi des traumatismes psychologiques et des préjudices moraux en raison des attaques répétées et de l'incapacité à fournir des soins adéquats. Les efforts d'atténuation ont été variés et ont reposé en grande partie sur des initiatives communautaires^6^.

L'étude fait partie du projet *Researching the Impact of Attacks on Healthcare^28^,* et a été réalisée en collaboration avec l'IMC, la Johns Hopkins University Bloomberg School of Public Health, l'Institut centrafricain des statistiques et des études économiques et sociales (ICASEES), le Ministère de la Santé et de la Population (MSP) et le Geneva Centre of Humanitarian Studies de l'Université de Genève.

## Méthodes

### Conception de l'étude

### L'étude présente le volet quantitatif d'un projet à méthodes mixtes visant à évaluer l'impact à long terme des attaques sur les soins de santé en RCA. Ce volet quantitatif se concentre sur l'évaluation des perturbations de service à l'aide des données sur les attaques identifiées et des données correspondantes des établissements de santé. Plusieurs approches analytiques ont été utilisées pour examiner l'évolution immédiate et à long terme des principaux indicateurs d'utilisation des soins de santé suite à des attaques contre des établissements de santé afin d'évaluer leur impact.

### Cadre et période de l'étude

Cette analyse s'étend aux zones de couverture des établissements de santé primaires, secondaires et tertiaires (statiques et mobiles) appuyés par IMC à Haute-Kotto, Ouaka et Vakaga, de janvier 2016 à décembre 2020 (Figure 1). Dans les préfectures, on estime que 46 % des établissements ne sont pas pleinement fonctionnels en raison de lacunes en matière de financement, d'infrastructures, de personnel, de fournitures et de médicaments.^27^ Malgré ces lacunes, le financement et le soutien externes aux établissements ne sont fournis que pour 46 % des établissements dans les préfectures. Outre l'IMC, le niveau de soutien et de couverture fourni par diverses ONG aux établissements de santé variait selon la préfecture et au fil du temps. Alors que de nombreuses ONG internationales et nationales étaient actives dans la Ouaka et la Haute-Kotto, le soutien dans la Vakaga était plus limité, seuls l'Organisation mondiale de la santé, le Comité international de la Croix-Rouge et Médecins sans frontières apportant un soutien aux services de santé de manière intermittente entre 2016 et 2020.


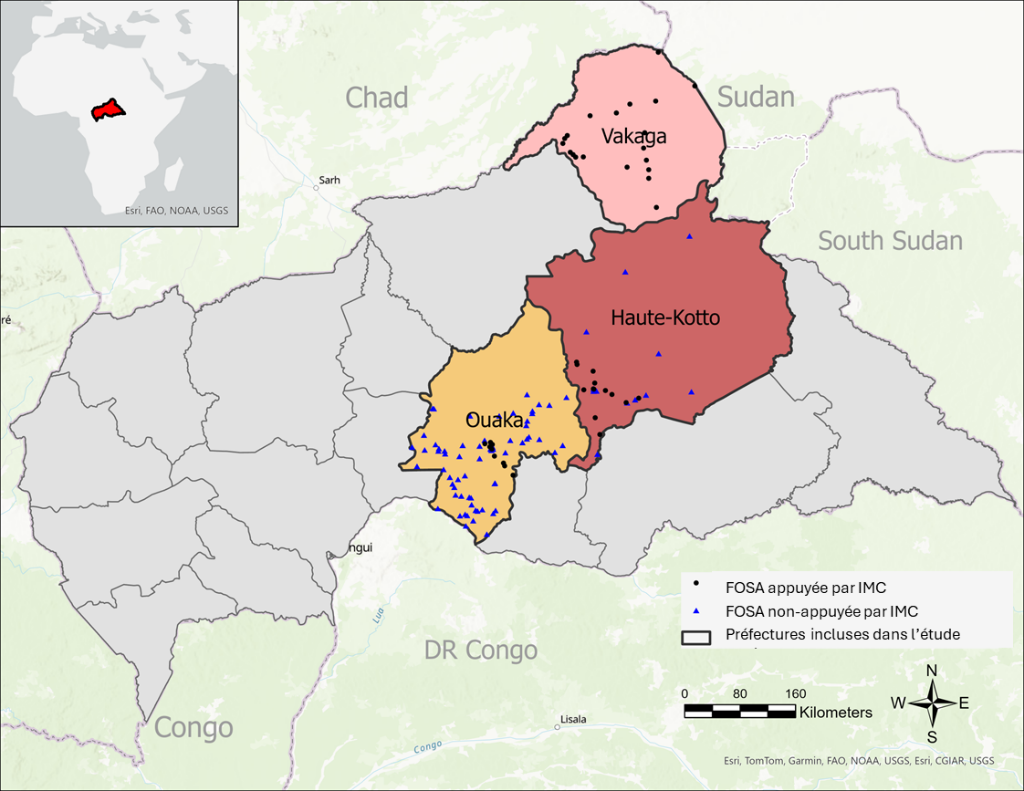


Figure 1 . Carte des préfectures incluses dans l'étude, avec l'emplacement des établissements de santé appuyés ou gérés par International Medical Corps entre 2016 et 2020 en République centrafricaine. Les établissements appuyés présentés sont ceux qui ont été appuyés en 2019 ; il y a eu une certaine variabilité dans la couverture d’appui au cours de la période 2016-2020.

### Définition de l'attaque sur les soins de santé

Nous avons défini une attaque contre les soins de santé comme "tout acte de menace, d'obstruction, de violence physique ou verbale à l'encontre du personnel de santé, des patients, des établissements de santé ou des transports sanitaires", conformément à l'Organisation mondiale de la santé (OMS), et s'étendant au personnel en service, aux patients, aux établissements ou aux transports protégés par le droit international humanitaire (DIH) pendant un conflit^29^. Les données sur les attaques contre les soins de santé proviennent de sources primaires et secondaires.

### Sources de données

#### Données primaires des attaques contre les soins de santé

Les entretiens avec les informateurs clés ont été menés en français, en anglais ou en sango, selon les préférences des personnes interrogées, par téléphone ou à l'aide de logiciels d'appels cryptés (Skype et Zoom), en trois phases (mai-juin 2022, décembre 2022-janvier 2023, et juin-août 2023). Les participants ont été sélectionnés à dessein sur la base de leur connaissance présumée des attaques contre les soins de santé dans les zones ciblées entre janvier 2016 et décembre 2020, et comprenaient des autorités administratives, du personnel de santé de première ligne, du personnel d'ONG et des autorités sanitaires au niveau régional et du district du MSP.

Les entretiens ont permis de recueillir des détails sur les attaques (date, lieu, type et description), l'impact perçu sur les installations, les services, la demande et l'utilisation des soins de santé, les mesures d'atténuation et tout facteur contextuel susceptible d'affecter les services et l'utilisation des soins de santé. L'étude étant axée sur l'impact des attaques plutôt que sur la responsabilité en vertu du DIH, il n'a pas été demandé aux informateurs clés d'identifier les auteurs, bien que certains aient fourni cette information.

Au total, 41 informateurs clés ont été interrogés, dont 36 ont été inclus dans l'étude. Les entretiens exclus concernaient des attaques survenues en dehors de la zone ou de la période d'étude. Les informateurs clés comprenaient des autorités administratives, du personnel de santé de première ligne, du personnel d'ONG et des autorités sanitaires régionales et de district du MSP. Des informations supplémentaires sur le profil des participants, ainsi que sur la formation et le processus d'entretien, sont disponibles dans la publication qualitative complémentaire, qui présente une analyse qualitative des entretiens avec des informateurs clés ^6^.

#### Données secondaires sur les attaques contre les soins de santé

Les données secondaires couvrant la période d'étude de janvier 2016 à décembre 2020 proviennent de quatre sources distinctes : 1. Insecurity Insight, 2. IMC, 3. Radio Ndeke Luka, et 4. une organisation anonyme.

La base de données Insecurity Insight est accessible au public^24^ et fournit des détails sur les attaques, notamment les dates, les descriptions, le niveau administratif de l'établissement, la localisation géographique approximative et, le cas échéant, le profil des auteurs. L'organisation anonyme a partagé une base de données contenant des variables similaires.

Les rapports des IMC sur la sécurité et des donateurs contenaient souvent des descriptions détaillées des attaques, y compris les dates, les descriptions, les effets immédiats et à long terme sur les opérations des IMC et les stratégies d'atténuation. Les rapports fournissaient également un contexte plus large du conflit dans les zones ciblées, y compris les violences non liées aux soins de santé, les déplacements de population, les activités politiques (par exemple, les élections, les visites de personnalités politiques dans la région, les négociations de paix), et d'autres facteurs susceptibles d'affecter l'accès aux soins de santé. Enfin, nous avons examiné de manière exhaustive les articles publiés par Radio Ndeke Luka^25^, une radio nationale et une plateforme de publication, afin d'extraire les données relatives aux attaques signalées dans la zone d'étude et au cours de la période considérée.

Nous avons exclu les données relatives aux attaques survenues en dehors de la période ou de la zone d'étude. Si le lieu d'une attaque n'était pas clair dans les autres sources, nous avons confirmé son occurrence dans la zone d'étude avec IMC. Lorsque les détails étaient incertains, nous avons demandé des informations supplémentaires à Insecurity Insight et les avons triangulées. La plupart de ces informations complémentaires provenaient de sources publiques, telles que des communiqués de presse et des articles d'actualité ; pour un petit nombre d'incidents, les sources étaient confidentielles, Insecurity Insight ne fournissant que des informations générales et dépersonnalisées.

#### Données des établissements de santé

Nous avons collecté chaque semaine des données secondaires auprès des établissements soutenus par l'IMC sur les services de santé, notamment le nombre de consultations externes, de premières visites de soins prénatals (CPN1), de doses de vaccin anti-rougeole (VAR1) administrées aux enfants de moins d'un an, d'accouchements dans les établissements de santé et d'hospitalisations. Les données ont été stockées et extraites de la plateforme de rapportage DHIS2 basée sur le cloud. Nous avons agrégé les données DHIS2 par mois.

### Analyse des données

#### Identification, triangulation et profilage des attaques contre les soins de la santé

Nous avons triangulé les sources de données primaires et secondaires afin de dédupliquer les attaques sur les soins de santé ou de compléter les informations manquantes, telles que les dates exactes des attaques lors des entretiens. L'ensemble de données final comprenait toutes les attaques mentionnées au moins une fois dans les ensembles de données primaires et secondaires, sans distinction selon qu'une attaque était mentionnée dans un seul ensemble de données ou dans plusieurs. Pour chaque attentat, la base de données finale comprenait Préfecture, type de lieu (établissement de santé, axe/route, zone générale), type d'établissement (hôpital, centre de santé, poste de santé, clinique mobile), lieu, date et catégorie d'attaque (pillage, menace, agression, meurtre, arrestation/détention, vol, blocus ou dommages aux infrastructures). Le cas échéant, nous avons enregistré plusieurs catégories d'attaques.

Nous avons généré des statistiques descriptives sur le nombre, le type et les caractéristiques des attaques afin de synthétiser les tendances en matière de nature et de répartition des violences contre les établissements de santé dans le temps et dans les zones géographiques. De plus, des informations clés sur des attaques et des établissements spécifiques sont fournies pour faciliter l'interprétation des résultats et garantir une bonne compréhension des conclusions dans le contexte opérationnel et sécuritaire où elles se sont produites. Les informations contextuelles sur les attaques proviennent des mêmes sources que celles qui ont permis de les identifier.

#### Niveau des établissements de santé

Nous avons classé les établissements en deux catégories : les hôpitaux et les établissements de référence, et les établissements de niveau primaire. Les hôpitaux et les structures de référence comprennent trois hôpitaux (un par préfecture) et deux centres de santé de référence (à Sikikide et Tiringoulou) à Vakaga. Les structures de niveau primaire comprennent les postes de santé, les unités médicales mobiles (MMU) et les centres de santé non référencés, car ils fournissent des ensembles de services plus similaires. Deux MMU, la PK3 àHaute-Kotto et la PK8 à Ouaka, fonctionnaient 24 heures sur 24 et 7 jours sur 7 avec du personnel d'urgence et du personnel IMC supplémentaire pendant la journée.

#### Approches analytiques

#### Nous avons appliqué quatre approches analytiques pour évaluer l'impact des attaques sur les services de santé : 1. Analyse visuelle des tendances d'utilisation des services avant et après chaque attaque ; 2. Calcul du changement immédiat de l'utilisation après une attaque ; 3. Analyse des séries chronologiques interrompues de l'utilisation des services avant et après chaque attaque ; et 4. Analyse de survie pour examiner le délai de déclaration du premier cas d'une maladie à potentiel épidémique, en l'occurrence la rougeole. Pour les trois premières méthodes, nous nous sommes concentrés sur les principaux services de soins de santé primaires pour lesquels nous disposions de données DHIS2 suffisantes : consultations externes, consultations prénatales, accouchements en établissement et vaccinations avec VAR1. Pour les hôpitaux et les établissements de référence, nous avons également inclus les hospitalisations comme indicateur d'intérêt. Pour l'analyse de survie, une épidémie nationale de rougeole s'est produite pendant la période d'étude et l'indicateur évalué était le délai de déclaration du premier cas de rougeole. Le tableau 1 présente un aperçu du nombre et du type d'établissements évalués pour chaque approche analytique.

Table 1. Formations sanitaires incluse dans chaque approche analytique, par prefecture

| Approach | Facility Level | Haute-Kotto | Vakaga | Ouaka |
| --- | --- | --- | --- | --- |
| Analyse visuelle des tendances | Hospital/ Référence | 1 | 3 | 1 |
|  | Primaire / MMU | 17 | 18 | 8 |
| Changement immédiat | Hospital/ Référence | 1 | 3 | - |
|  | Primaire / MMU | 11 | 7 | 2 |
| Analyse des series chronologiques interrompues | Hospital/ Référence | - | 1 | - |
|  | Primaire / MMU | 6 | - | 1 |
| Analyse de survie | Hospital/ Référence | - | 3 | - |
|  | Primaire / MMU | - | 17 | - |
| *Toutes les structures sanitaires incluse ont été soutenues par International Medical Corps de manière continue ou intermittente de 2016 à 2020.*  *MMU – Mobile Medical Unit* | | | | |

#### Analyse visuelle des tendances d'utilisation des services avant et après chaque attaque

#### Nous avons réalisé une analyse visuelle en représentant graphiquement l'utilisation mensuelle des services pour chaque établissement, avant et après chaque attaque. Cette approche a fourni un moyen simple et accessible d'évaluer l'évolution de l'utilisation au fil du temps, notamment la direction et l'ampleur des changements pour des services spécifiques au sein de chaque établissement. Ces graphiques nous ont permis d'examiner des tendances qui ne pouvaient être résumées de manière significative par une seule mesure. La visualisation nous a permis d'évaluer les impacts spécifiques aux services et à l'échelle de l'établissement au fil du temps, en mettant en évidence les variations de perturbation et de reprise selon les contextes.

Les résultats sont présentés pour trois attaques qui capturent les types d’impact observés dans les installations.

#### Calcul du changement immédiat après une attaque

Nous avons agrégé les données DHIS2 par mois. Pour quantifier le changement immédiat des indicateurs clés après chaque attaque, nous avons calculé le pourcentage de changement en comparant les données du mois précédant l'attaque à celles du mois suivant. Par exemple, si une attaque a eu lieu en mars 2020, nous avons calculé la variation proportionnelle des indicateurs entre février et avril 2020.

Les attaques multiples survenues au cours de mois consécutifs dans la même installation ont été considérées comme un cas unique et la variation de la proportion a été calculée à partir du mois précédant la première attaque jusqu'au mois suivant la dernière. Par exemple, si des attaques ont eu lieu en mars et avril 2020 dans la même installation, la variation de la proportion a été calculée de février à mai 2020.

L'analyse a été limitée aux attaques survenues dans l'établissement ou impliquant du personnel en service, dont le mois et l'année de l'attaque étaient connus et pour lesquelles les données de l'établissement étaient disponibles pour la période requise. Les attaques signalées au cours des premier et dernier mois de la période d'étude (janvier 2016 et décembre 2020) ont été exclues en raison de l'absence de données avant et après l'attaque. Lorsque les valeurs avant l’attaque étaient nulles en raison de données probablement manquantes (par exemple, des échecs de transmission de rapport ou une perte d’enregistrement pendant le pillage), nous avons remplacé les valeurs du mois précédent sur la base de la documentation organisationnelle. De 127 attaques identifiés, l'analyse finale a porté sur neuf attaques contre des hôpitaux ou des établissements de niveau de référence, et sur 31 attaques contre des établissements de niveau primaire.

*Analyse des séries chronologiques interrompues de l'utilisation des services*

Pour estimer les effets à long terme des attaques, nous avons utilisé une approche de séries chronologique interrompues (SCI) en utilisant les données avant l’attaque pour comparer les valeurs attendus et observés dans les 12 mois suivant l’attaque. Pour chaque attaque et indicateur d'intérêt, nous avons ajusté un modèle additif généralisé, en supposant une structure de corrélation AR1 et une distribution binomiale négative. Le modèle comprenait des termes pour la tendance mensuelle, ainsi que pour le changement immédiat du niveau et le changement de tendance mensuelle à la suite d’une attaque. La saisonnalité a été modélisée à l'aide de quatre splines de régression cubique cycliques. Le modèle a été ajusté à l'aide du package mgcv de R^31^.

Pour estimer la différence entre les valeurs observées et les valeurs contrefactuelles (valeurs attendues si l'attaque n'avait pas eu lieu), nous avons généré 1000 tirages à l'aide d'une procédure bootstrap paramétrique^32^ à partir d'un modèle dans lequel les coefficients d'attaque ont été fixés à 0. Pour les 12 mois suivant une attaque, nous avons généré la médiane et les intervalles de prédiction à 95 % pour les différences absolues et proportionnelles entre les valeurs observées et les valeurs contrefactuelles. La différence proportionnelle a été calculée comme la différence absolue entre les valeurs observées et les valeurs contrefactuelles, divisée par les valeurs contrefactuelles.

Dans la mesure du possible, nous avons comparé les changements survenus avant et après l’attaque avec les changements survenus dans un établissement témoin, défini comme un établissement fournissant des services similaires dans une zone géographique similaire et dont la tendance séculaire commune est attendue. Pour les établissements de niveau de référence, nous avons pris l'établissement de niveau de référence le plus proche, situé dans la même préfecture que l'établissement attaqué. Pour les établissements de niveau primaire, nous avons pris les établissements situés sur le même axe périphérique, étant donné que différents groupes armés contrôlaient différents axes au cours de la période. Les établissements ayant subi une attaque dans la période précédant ou dans les douze mois suivant l'attaque en question ont été exclus en tant que témoins.

Les modèles ont été ajustés indépendamment pour les établissement attaqué et les établissements témoins, la date d'interruption étant fixée à la date de l'attaque. Pour permettre la comparaison, nous avons inclus dans chaque modèle un décalage égal au nombre moyen de consultations de l’établissement avant l’attaque. Les modèles ajustés et les contrefactuels sont présentés sous forme de graphiques afin de permettre une comparaison visuelle des tendances.

En raison de critères d'exclusion stricts (nécessitant au moins 12 mois de données avant et après l'attaque et aucune autre attaque au cours de cette période), seul un petit sous-ensemble des formations et d'attaques a été inclus ; à notre connaissance, ces formations ne présentaient aucune donnée manquante, car les zéros post-attaque ont été vérifiés par le biais de rapports organisationnels comme de véritables suspensions de service plutôt que comme des valeurs manquantes.

*Analyse de survie du premier cas signalé d'une maladie à potentiel épidémique (rougeole)*

Lorsque le système de santé est perturbé par des attaques contre les soins de santé, le risque de maladies évitables par la vaccination et à potentiel épidémique augmente, contribuant potentiellement à une mortalité évitable. Entre février 2019 et janvier 2020, la RCA a signalé 3653 cas de rougeole, avec une épidémie déclarée en 2019 dans cinq districts^33^. Après l'épidémie, nous avons évalué si les attaques contre les soins de santé étaient associées au délai de déclaration du premier cas de rougeole par l'établissement. Nous avons analysé les données au niveau de l'établissement de santé, en définissant l'exposition comme toute attaque contre les soins de santé en 2018 (l'année précédant l'épidémie). Aucun établissement soutenu par l'IMC n'a déclaré de cas de rougeole en 2020, probablement en raison de lacunes dans la surveillance des établissements ou dans les données de déclaration, puisque les cas de rougeole à Vakaga ont été signalés ailleurs^34^. Alors l'inclusion exigeait qu'un établissement ait au moins 6 mois de déclaration ou au moins un cas de rougeole en 2019. Nous avons exclu les établissements qui n'étaient pas soutenus par l'IMC pour les services de santé pédiatriques au cours de la période d'étude. En raison de la rareté des établissements répondant aux critères d'inclusion à Haute-Kotto et Ouaka, nous avons limité les analyses à la préfecture de Vakaga.

Afin de distinguer les vrais zéros des données manquantes, nous avons utilisé les consultations ambulatoires hebdomadaires, déclarées sur les mêmes formulaires, comme indicateur indirect des déclarations des établissements, en considérant les semaines sans consultation comme non déclarées et en excluant les établissements pour lesquels plus de la moitié des semaines manquaient. Les cas manquants ou non déclarés étaient relativement limités dans les autres établissements, comme le montre la figure S1 ; étant donné que les lacunes étaient souvent signalées rétroactivement et que la rougeole est une maladie à déclaration obligatoire, nous avons considéré qu'il était peu probable que des cas suspects ou confirmés ne soient pas déclarés. Nous avons donc traité le premier cas déclaré comme le véritable premier cas, ce qui est corroboré par la cohérence avec les rapports mensuels internes.

Nous avons effectué une analyse de survie comparant le temps écoulé avant le premier cas de rougeole signalé entre les établissements exposés et les établissements non exposés. Nous avons émis l'hypothèse que les établissements ayant subi une attaque avaient une couverture VAR plus faible, ce qui entraînait une augmentation du nombre d'enfants sensibles et une introduction plus précoce de la rougeole. Les tests statistiques et les courbes de survie ont été générés à l'aide des packages R survival^35^ et ggsurvfit^36^, respectivement.

## Résultats

### Profile des attaques identifiées

#### Nombre et source des attaques

Nous avons identifié beaucoup plus d'attaques que celles rapportées dans les bases de données existantes, avec 127 attaques rapportées sur cinq ans dans les trois préfectures (Tableau 2). La majorité des attaques ont été identifiées à partir des rapports et communications internes de l'IMC (72 attaques, 57%), suivies par des entretiens avec des informateurs clés (47 attaques, 37%) (Tableau 2). La plupart des attaques ont été signalées par une seule source (110 attaques, 87%), tandis que 13% ont été signalées par deux sources de données. Seules cinq attaques pertinentes (4%) sont apparues dans Radio Ndeke Luka.

*Tableau 2. Source des données relatives aux attaques. Le nombre total correspond au nombre dédupliqué d'attaques identifiées.*

| **Source des données sur les attaques** | **Ensemble,** N = 127^1^ |
| --- | --- |
| Rapports et communications internes du CIM | 72 (57%) |
| Entretiens avec des informateurs clés | 47 (37%) |
| Aperçu de l'insécurité | 13 (10%) |
| Source anonyme | 6 (5%) |
| Radio Ndeke Luka | 5 (4%) |
| ^1^n (%) | |

#### Date et lieu des attaques

La figure 2 présente le nombre et le type d'attaques contre la santé, par mois, de janvier 2016 à décembre 2020 dans les trois préfectures. La plus forte concentration d'attaques a été observée dans la Haute-Kotto, notamment entre mi-2017 et mi-2018, où la plupart des attaques ont concerné des structures sanitaires. En revanche, les attaques dans la Ouaka et la Vakaga ont été moins nombreuses et plus sporadiques. La répartition et la distribution des attaques semblent refléter une dynamique de conflit plus large : la plupart des attaques en Haute-Kotto ont eu lieu en 2017-2018, tandis qu'en Vakaga, les attaques ont été plus fréquentes en 2019-2020. Dans l'ensemble des préfectures, 61 % des attaques identifiées ont eu lieu dans un établissement de santé ou ont impliqué du personnel en service, le reste se produisant le long d'axes périphériques (17 %) ou dans des zones générales (22 %). Les attaques sur les axes impliquaient principalement des unités médicales mobiles (UMM) circulant sur les routes, tandis que les attaques de zone générale comprenaient des attaques telles que le pillage des bases de l'IMC et l'obstruction des équipes de l'IMC ou des patients par des groupes armés.


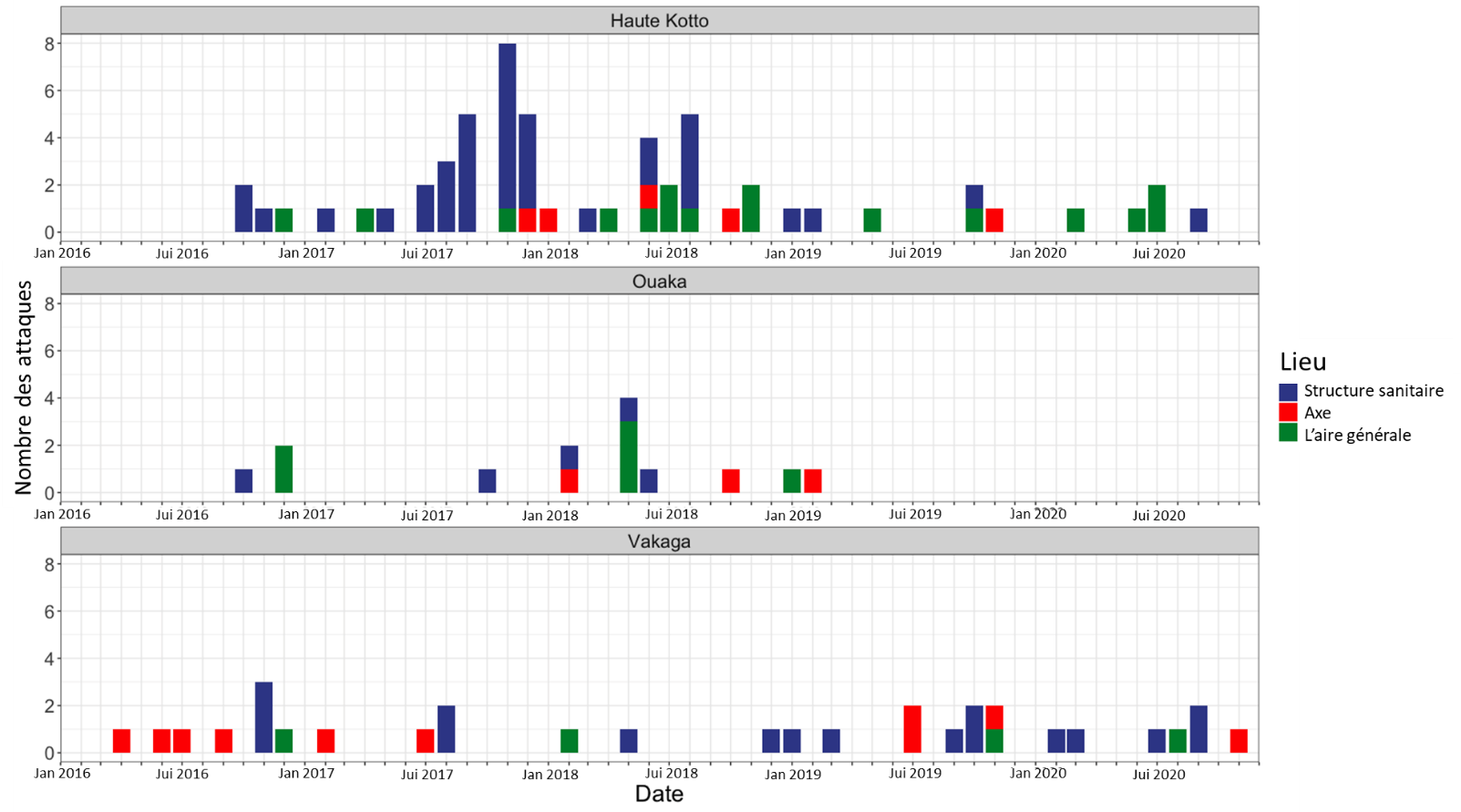


*Figure 2. Attaques identifiées contre les soins de santé dans les structures de santé, les axes périphériques, et les aires générales appuyés par ou avec la présence d’International Medical Corps, de 2016 à 2020, par préfecture et par année.*

#### Types des attaques Certains établissements ont subi plusieurs attaques au cours de la période étudiée. Par exemple, dans un hôpital, nous avons identifié dix attaques distinctes, y compris l'occupation, les menaces, les agressions physiques, les coups de feu et les affrontements. Sur les 127 attaques, 36 (28,3 %) relevaient de plusieurs catégories. Parmi les catégories d'attaques, l'enlèvement ou la tentative d'enlèvement de biens est la plus fréquente (26 ,7 %), suivie par les menaces (18,2 %) et le pillage (16,5 %) (Tableau 3). Des violences physiques ou sexuelles – y inclus des agressions, des meurtres, et des arrestations, des détentions, des actes de torture ou des enlèvements - ont été constatées dans respectivement 10,2 %, 7,2 % et 5,7 % des attaques. Au total, une forme de violence physique ou sexuelle a été signalée dans 23,3 % des agressions, dont 13 meurtres.

#### Tableau 3. Attaques contre les soins de santé signalées, par catégorie d'attaque et par préfecture sanitaire. Un seul cas d'attaque (N=127) peut être classé dans plusieurs catégories d'attaques. La catégorie "enlèvement ou tentative d’enlèvement de biens" comprend le vol ou la confiscation de biens, le brigandage ou la tentative de brigandage, et le cambriolage ou la tentative de cambriolage.

| **Catégorie d'attaque** | **Ensemble,** N = 176^1^ | **Haute-Kotto**, N = 75^1^ | **Ouaka**, N = 42^1^ | **Vakaga**, N = 59^1^ |
| --- | --- | --- | --- | --- |
| Enlèvement ou tentative d'enlèvement des biens | 47 (27%) | 20 (27%) | 9 (21%) | 18 (31%) |
| Menace | 32 (18%) | 19 (25%) | 2 (4.8%) | 11 (19%) |
| Pillage | 29 (16%) | 9 (12%) | 8 (19%) | 12 (20%) |
| Agression physique ou sexuelle | 18 (10%) | 5 (6.7%) | 7 (17%) | 6 (10%) |
| Meurtre | 13 (7.2%) | 5 (6.7%) | 6 (14%) | 2 (3.4%) |
| Arrestation, détention, torture ou enlèvement | 10 (5.7%) | 4 (5.3%) | 1 (2.4%) | 5 (8.5%) |
| Intrusion, occupation ou affrontements | 9 (5.1%) | 4 (5.3%) | 5 (12%) | 0 (0%) |
| Blocage d'une ambulance sur la route / Extorsion | 8 (4.5%) | 5 (6.7%) | 1 (2.4%) | 2 (3.4%) |
| Attentats à la bombe, coups de feu ou incendies criminels | 5 (2.8%) | 3 (4.0%) | 2 (4.8%) | 0 (0%) |
| Dommages | 5 (2.8%) | 1 (1.3%) | 1 (2.4%) | 3 (5.1%) |
| ^1^n (%) | | | | |

Analyse visuelle des tendances d’utilisation des services avant et après l’attaque

L'évolution de l'utilisation des services variait considérablement selon les services, les établissements et le temps. Nous avons observé trois tendances généraux d'utilisation des services après une attaque : fermetures complètes et prolongées des établissements (par exemple, le poste de santé de Ngoubi), interruptions à long terme de services spécifiques (par exemple, la perte des services de vaccination de routine au poste de santé d'Irabanda) ou aucun interruption ou interruptions à court terme (par exemple, le poste de santé de Madomale). La gravité de ces schémas variait considérablement, reflétant la diversité des impacts des attaques sur la prestation de services dans les établissements.

Au poste de santé de Ngoubi, un groupe armé a pillé l'établissement en novembre 2017, provoquant la fuite de tout le personnel de santé et l'interruption des services pendant plus d'un an. La cartographie visuelle des données de service du poste de santé de Ngoubi (Haute-Kotto) montre l'arrêt des services, des consultations externes, des CPN1, des accouchements et de la vaccination VAR1 (Figure 3A). En août 2018, le poste de santé d'Irabanda a subi de multiples attaques au cours desquelles le directeur de l'établissement a été assassiné dans une embuscade et l'établissement a été pillé. La cartographie n'illustre aucun changement perceptible dans l'utilisation des services externes, des CPN1 ou des accouchements après la série d'attaques. Cependant, l'utilisation du VAR1 est tombée à zéro et est restée suspendue, à l'exception d'une campagne de vaccination début 2020 (Figure 3B). L'arrêt des services de vaccination était dû à des failles dans la chaîne du froid, les équipes du district sanitaire et de l'IMC n'ayant pas pu livrer les vaccins en raison de protocoles de sécurité révisés. En revanche, pour le poste de santé de Madomale, aucun changement n'a été observé dans le schéma d'utilisation des services avant et après l'attaque d'octobre 2017 (figure 3C). Au contraire, les fluctuations d'utilisation des services sont similaires avant et après l'attaque.

*Figure 3. Exemples illustratifs de changements dans l'utilisation des services dans trois postes de santé des préfectures de Haute-Kotto et de Ouaka à la suite d'une attaque contre les soins de santé.* ***A.*** *Arrêt complet des services fournis/utilisés. Le panneau montre la tendance mensuelle pour les consultations externes, la première visite de consultation prénatale (CPN1), les accouchements et la première dose de vaccin anti-rougeole (VAR1) fournis par le Poste de Santé Ngoubi (préfecture de Haute-Kotto) pillé en novembre 2017.* ***B.*** *Changement dans certains services sur une période plus longue. Le panneau montre les tendances mensuelles des services de santé à Poste de Santé Irabanda (préfecture de Haute-Kotto) après le pillage d'août 2018. Une campagne de vaccination de masse a eu lieu en avril 2020.* ***C.*** *Pas de changement observable, ou très courte durée de changement dans l'utilisation des services. Le panneau montre les tendances mensuelles des services de santé dans la Poste de Santé Madomale (préfecture de Ouaka) après le pillage d'octobre 2017. * La ligne pointillée rouge représente l'attaque.*

**
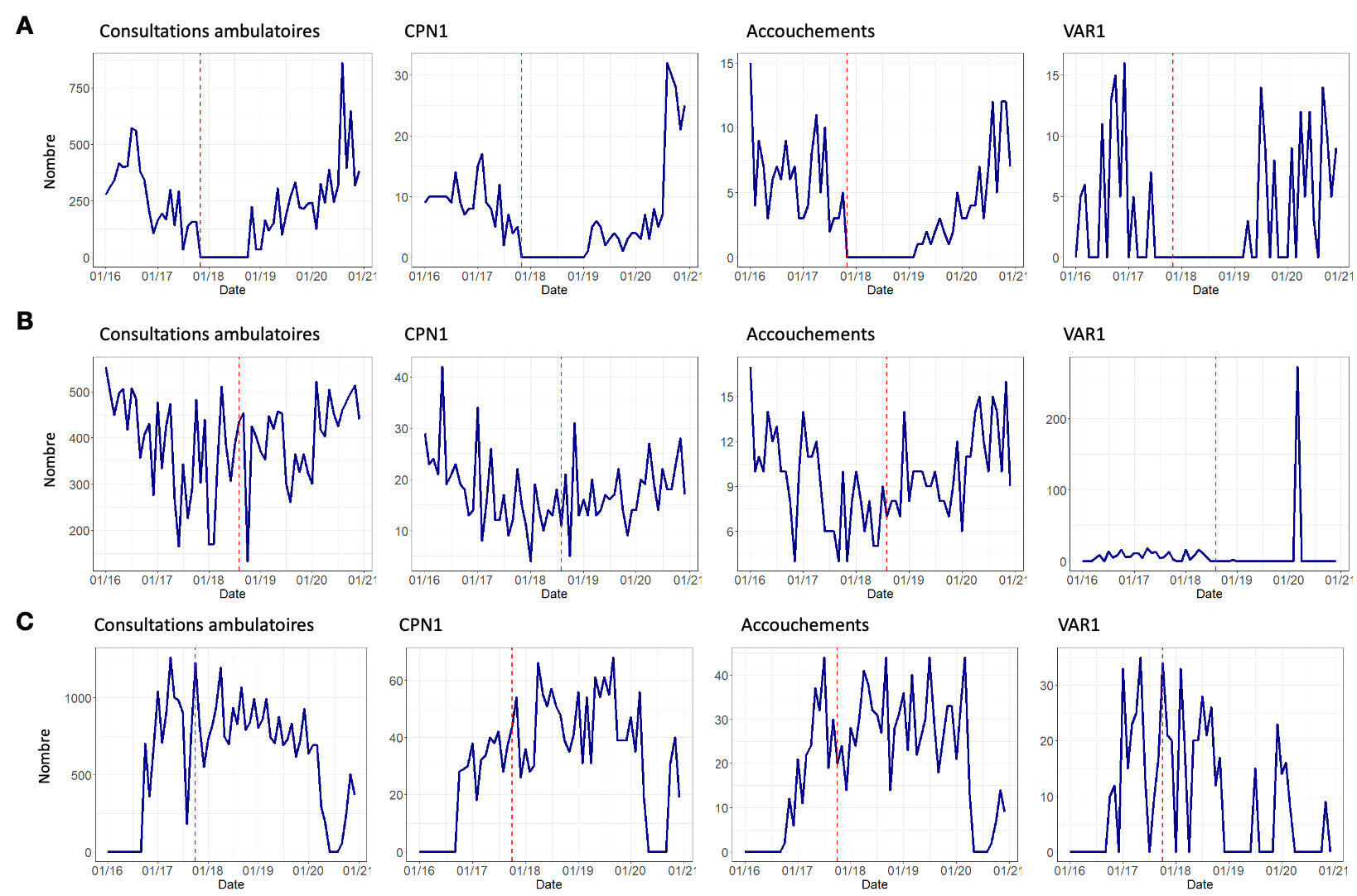
**

### Estimation de changement immédiate de l'utilisation des services à la suite d'une attaque contre les soins de santé

Comme pour les changements dans le temps, les résultats concernant les effets immédiats varient selon les services, le niveau de l'établissement et le temps (figure 4). Les changements proportionnels allaient de -100 % (arrêt complet des services) à une augmentation de 655 % par rapport aux niveaux antérieurs à l'attaque. Pour les hôpitaux et les établissements de référence, nous n'avons observé aucune diminution ≥25% dans les accouchements, alors que dans deux des trois attaques, les niveaux de VAR1 ont diminué de 50% et de 100%. Les changements dans les hospitalisations, les consultations externes et les CPN1 ont été mitigés.

Pour les établissements de niveau primaire, le VAR1 a diminué de plus de 25 % pour trois des cinq attaques. L'évolution des consultations, des CPN1 et des accouchements n'a pas montré de tendance claire. Pour de nombreuses attaques, les indicateurs ont évolué dans le même sens et avec la même ampleur (par exemple, augmentation ou diminution de 50 % des accouchements, des CPN1 et des consultations), mais ce n'était pas toujours le cas.

*Figure 4. Changement immédiat dans les services à la suite d'une attaque contre les soins de santé, par service de santé fourni. Le changement immédiat est calculé comme la différence de proportion des indicateurs d'intérêt dans le mois suivant l'attaque par rapport au mois précédant l'attaque. Les étiquettes indiquent l'établissement auquel l'attaque est associée et le mois au cours duquel l'attaque a eu lieu. Pour les cas d'attaques multiples au cours des mois suivants, la date indique le mois de la première attaque.*


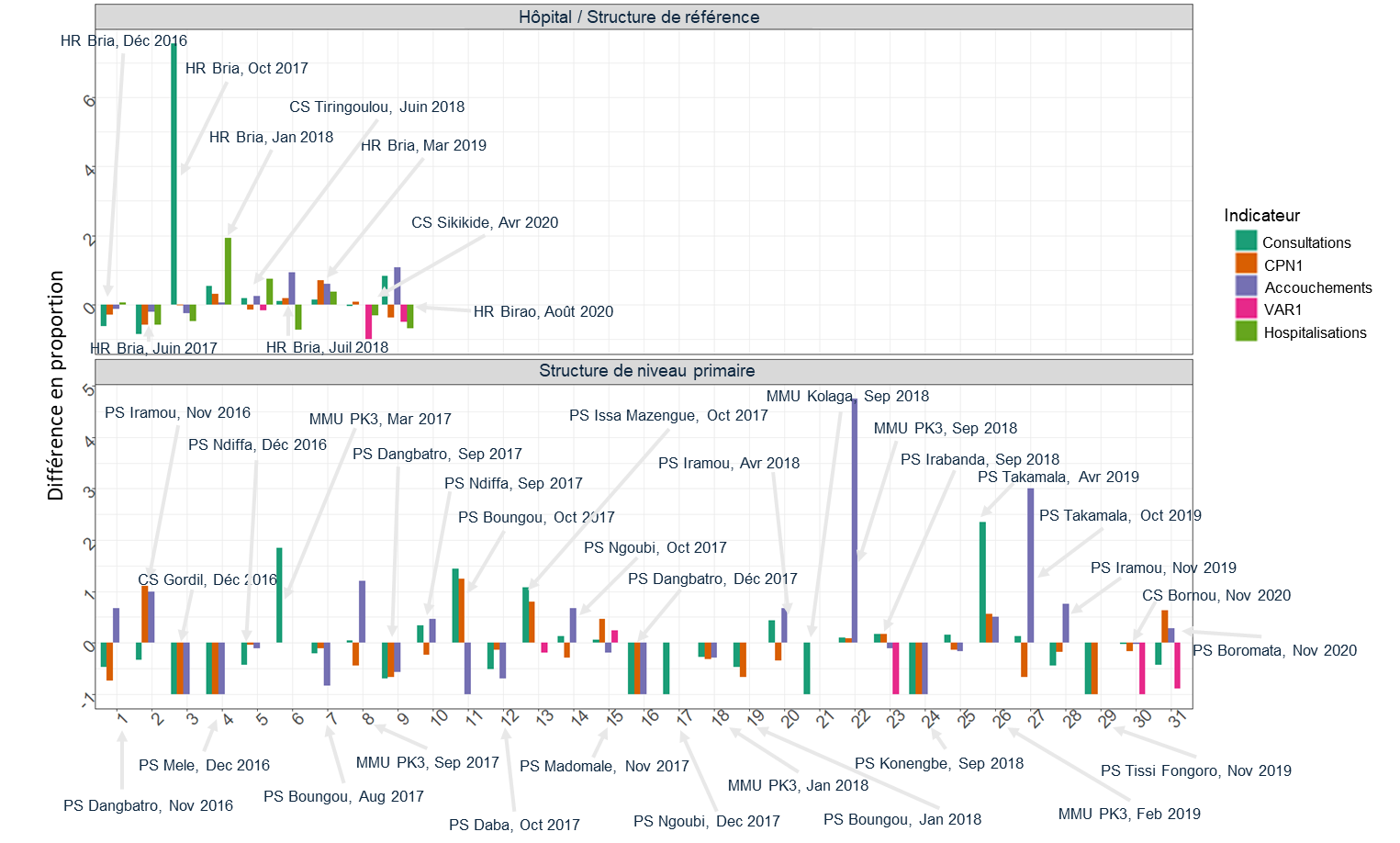


### Ces estimations de changements immédiats ne doivent cependant pas être interprétées comme des impacts directs des attaques, car d'autres facteurs peuvent expliquer les différences observées. Par exemple, l'augmentation de 655 % des consultations externes a été observée à HR Bria, à la suite de multiples attaques de juillet à septembre 2017. Au cours de cette période, l'insécurité, les déplacements et les affrontements étaient généralisés dans la région, tandis qu'un accord de cessez-le-feu en septembre 2017, accompagné d'activités de consolidation de la paix, pourrait avoir augmenté la demande et l’accès de soins de santé en octobre 2017. De plus, le mois « pré-attaque », mai 2017, a été caractérisé par une violence intense, y compris des déplacements de population à grande échelle, ce qui suggère que l'augmentation observée de 655 % reflète des tendances séculaires plus larges plutôt que les effets de l'attaque seule.

### Analyse des séries chronologiques interrompues d’utilisation des services

Nous avons analysé huit structures de santé disposant d'au moins 12 mois de données avant et après l'attaque, dont six à Haute-Kotto, un à Ouaka et un à Vakaga. L'un d'entre eux était un établissement de référence et sept étaient des établissements de niveau primaire. De plus amples détails sur la sélection des établissements et l'ajustement des modèles sont présentés dans le supplément (annexe 2).

Dans quatre formations sanitaires (Madomale, Konengbe, Boungou et Tiringoulou), la médiane des consultations observées après l'attaque était inférieure au contrefactuel, les postes de santé de Ngoubi et de Boungou connaissant une réduction de plus de 25 % (Figure 5). En revanche, trois formations sanitaires (Issa Mazengue, Irabanda et Daba) ont connu une augmentation de plus de 25 % par rapport au scénario contrefactuel. Une structure, Ngoubi, a été complètement fermée pour l'année, avec une réduction de 100 % des consultations, des CPN1 et des accouchements. Dans l'ensemble, il n'y a pas eu de direction cohérente de changement entre les indicateurs.


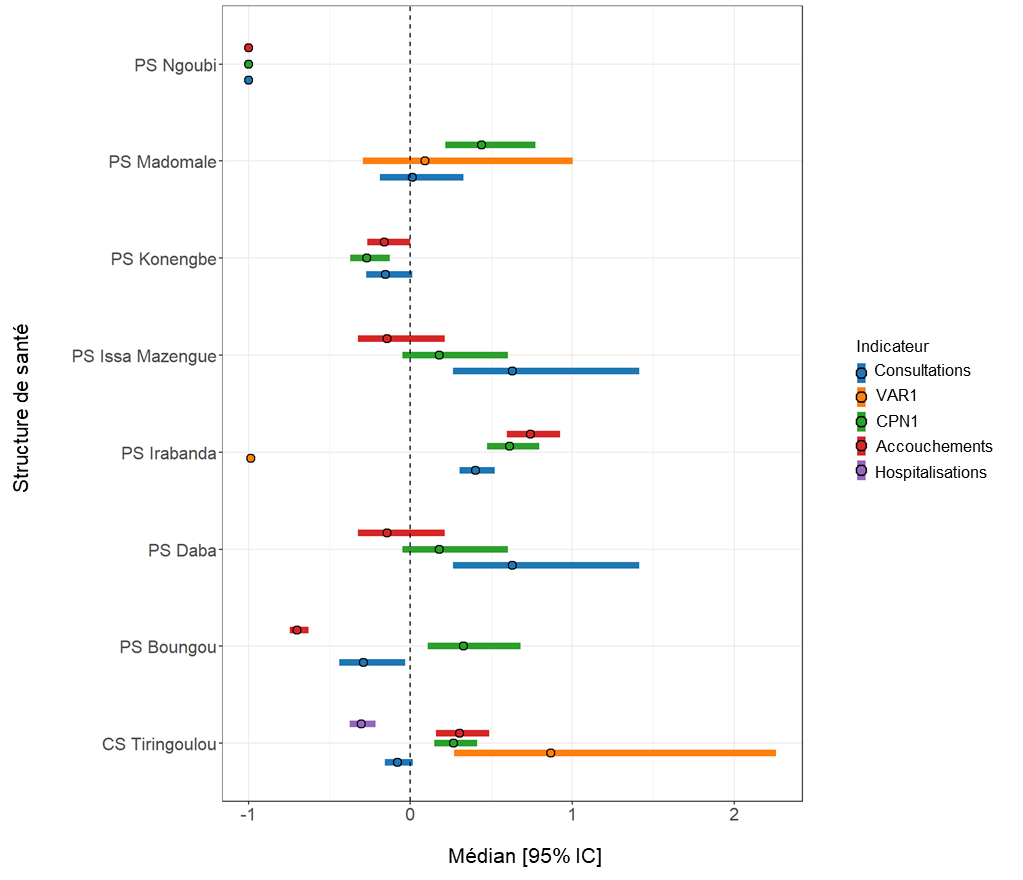


*Figure 5. Diagramme de forêt présentant la médiane et les intervalles de confiance à 95 % pour la différence de proportion entre les niveaux observés des indicateurs d'intérêt et les niveaux attendus (contrefactuels) dans les 12 mois suivant une attaque contre les soins de santé. Le point noir représente la médiane et la barre indique l'étendue des intervalles de confiance.*

Nous avons considéré trois exemples spécifiques pour illustrer les résultats. L’attaque contre le poste de santé de Ngoubi en novembre 2017 est à nouveau illustrée dans la figure 6A. L'attaque a été associée à une perte estimée à plus de 1000 consultations, avec une perte médiane de 45 accouchements et 71 visites CPN1. Les principaux obstacles à la reprise des services étaient la perte de matériel et de fournitures, le déplacement de la population et la crainte du personnel de santé de reprendre les activités tant que la population n'était pas revenue. Fin 2018, le MSP et l'IMC ont mené des visites exploratoires, des activités de sensibilisation et des efforts pour encourager le personnel à revenir. Les activités ont repris en février 2019.

En août 2018, malgré les attaques contre le poste de santé d'Irabanda qui ont inclus des assassinats et des pillages, nous n'avons pas observé de diminution des consultations, des ANC1 ou des accouchements, bien que comme décrit dans l'analyse visuelle, les vaccinations MCV1 aient cessé en raison de perturbations dans la chaîne du froid (Figure 6B).

Enfin, le centre de santé de Tiringoulou, une structure de référence, a été attaqué en mai 2018, les auteurs neutralisant trois membres du personnel et pillant la structure. Malgré cela, les dégâts se sont limités à la perte de médicaments et de fournitures telles que des matelas, sans destruction structurelle. La proximité d'une piste d'atterrissage a permis une chaîne d'approvisionnement stable et un rétablissement rapide. Les données post-attaque n'ont montré qu'une légère baisse des consultations et des hospitalisations, ainsi qu'une légère augmentation des consultations prénatales (CPN1), des accouchements assistés et des vaccinations contre le VAR1 (Figure 6C).


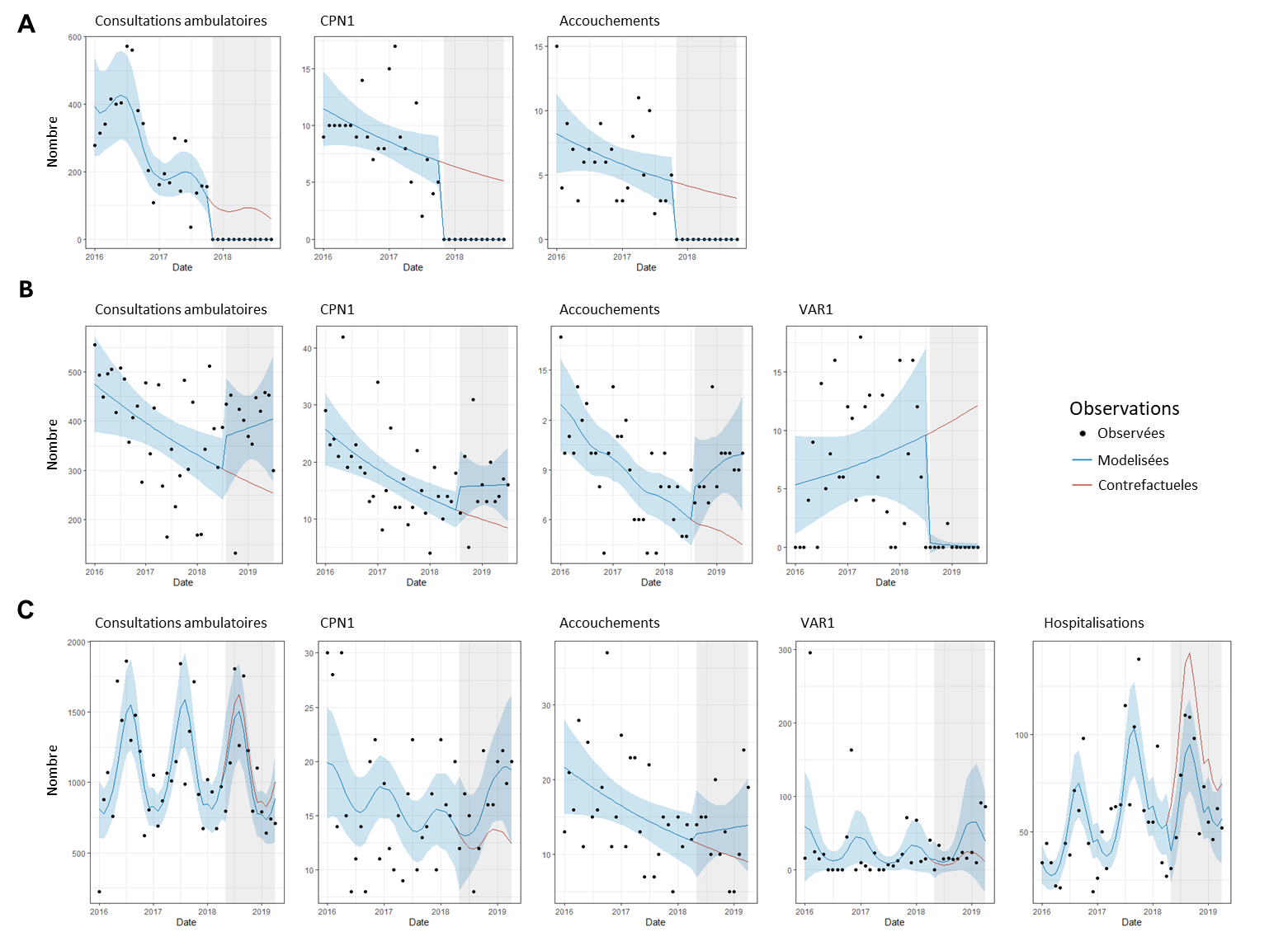


*Figure 6. Séries chronologiques interrompues pour trois établissements de santé ayant subi des attaques entre 2016 et 2020. Les panneaux affichent les valeurs observées pour les consultations, les CPN1, les accouchements, le VAR1 et les hospitalisations. La ligne bleue et le ruban indiquent la médiane et les intervalles de confiance du modèle; la ligne rouge est la valeur médiane contrefactuelle pour la période post-attaque.* ***A****. Poste de Santé Ngoubi, pillé en novembre 2017.* ***B****. Poste de Santé Irabanda, qui a subi un pillage, un vol et un meurtre en août 2018.* ***C.*** *Centre de Santé Tiringoulou (structure de référence), pillé en mai 2018.*

L'analyse a été limitée par la difficulté à trouver des structures de témoin, en particulier à Haute-Kotto, où la plupart des structures ont subi des attaques en 2017-2018. Pour le poste de santé de Boungou, où un agent de santé a été assassiné en juillet 2017, nous avons identifié un centre de santé pour servir de témoin plausible. Alors que Boungou a connu une baisse des consultations externes et des accouchements dans les douze mois suivant l'attaque, la même baisse n'a pas été observée dans la structure de témoin (Figure 7).


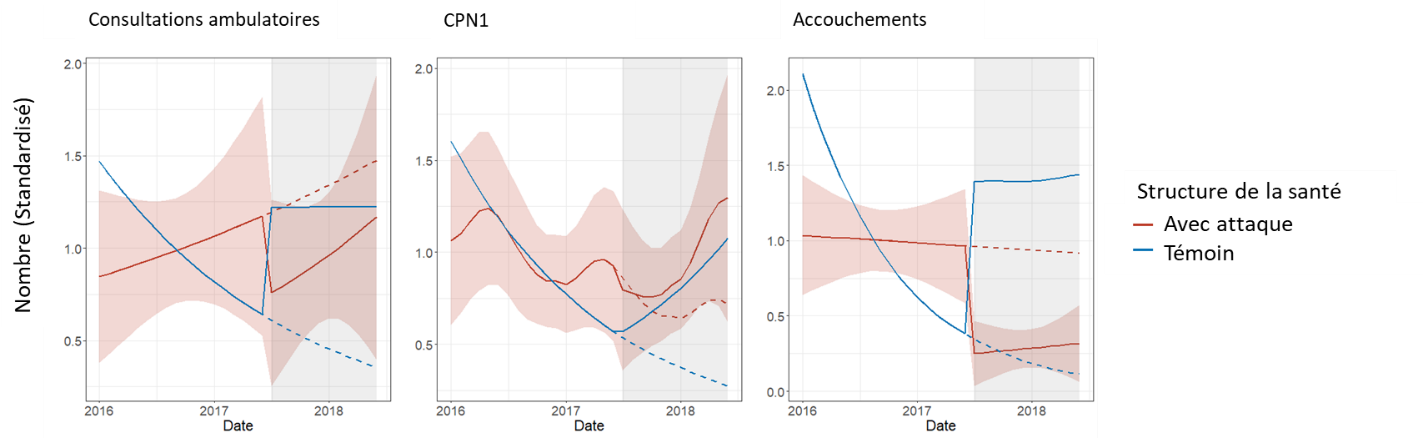


*Figure 7. Série chronologique interrompue pour Poste de Santé Boungou, qui a connu un meurtre du personnel de la formation sanitaire en juillet 2017, et formation de témoin dans la préfecture de Haute-Kotto. La structure de témoin n'a pas connu d'attaque pendant cette période. La ligne en continue représente le modèle aux données observées pendant la période post-attaque, tandis que la ligne pointille est le contrefactuel.*

### *Comparaison du délai de survenue du premier cas de rougeole dans les établissements ayant subi des attaques et dans ceux qui n'en ont pas subi dans la préfecture de Vakaga*

Parmi les vingt structures de santé analysés, neuf ont déclaré au moins un cas de rougeole pendant l'épidémie de 2019. Un diagramme de nageur décrivant le temps écoulé avant le premier cas de rougeole est présenté dans le supplément (Figure S1). Le test du log-rank pour la différence dans les courbes de survie suggère une différence dans le temps écoulé avant le premier cas de rougeole (p<0,001) (Figure 8) ; cependant, les résultats doivent être interprétés avec prudence en raison du petit nombre des structures de santé exposés (3 attaqués en 2018), par rapport aux structures non exposés (17 sans attaques).


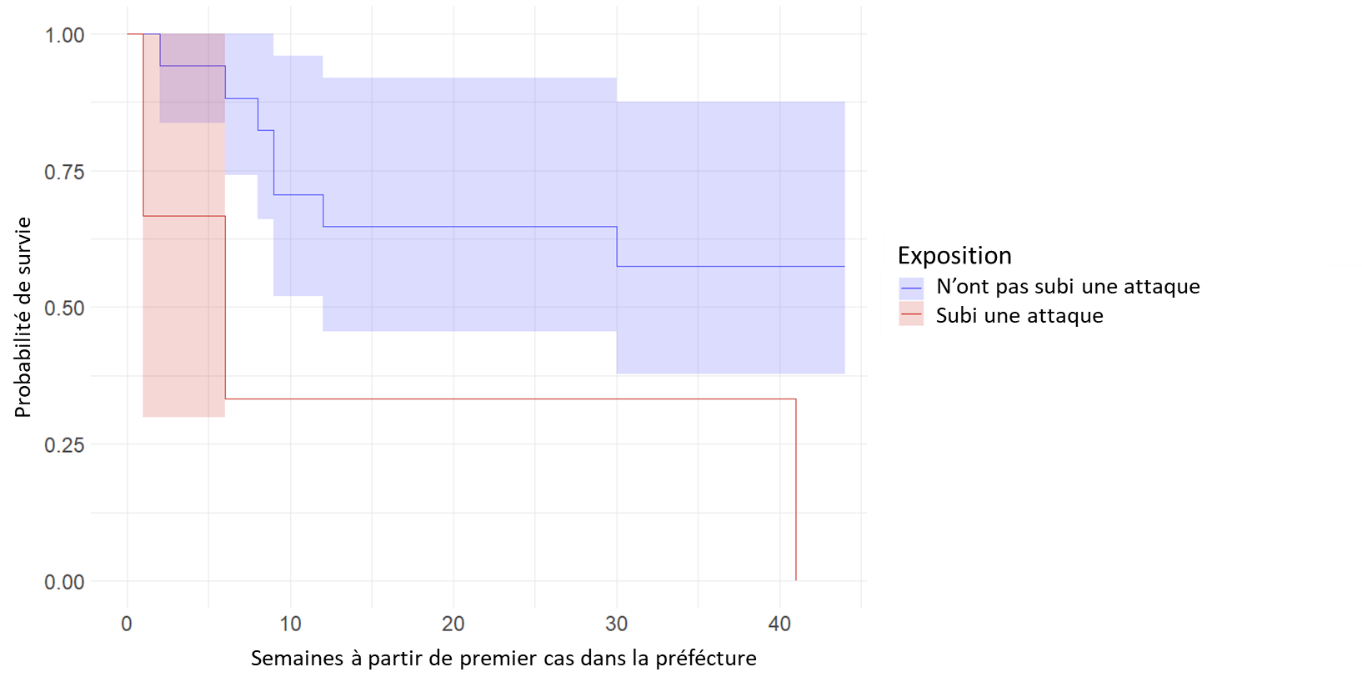


*Figure 8. Courbes de survie des structures de santé ayant subi des attaques et de ceux qui n'en ont pas subi dans la préfecture de Vakaga. Le résultat est défini comme la déclaration d'un cas de rougeole ; l'exposition est définie comme une attaque contre les soins de santé identifiée en 2018. L'origine est le moment de la déclaration du premier cas de rougeole dans la préfecture.*

## Discussion

Impacts variables des attaques sur les soins de santé

La fréquence et la répartition des attaques ont coïncidé avec les périodes de conflit intense dans les préfectures, avec un pic en Haute-Kotto entre 2017 et 2018, dans la Vakaga en 2016 et 2019-2020, et dans la Ouaka en 2018 et 2020. Ces tendances reflètent l'influence des dynamiques de conflit locales sur le ciblage des soins de santé et peuvent être influencées par la présence de groupes armés, l'évolution des lignes de front et le contrôle politique du territoire. Les différences régionales en termes de qualité des soins de santé, de caractéristiques de la population et de contexte, ainsi que les variations dans le type, l'intensité et la récurrence des attaques, influencent le degré de perturbation observé entre les sites.

Certains établissements ont subi de multiples attaques au cours de la période d'étude, compliquant davantage le rétablissement et la continuité des services. Un hôpital a été victime de dix incidents distincts, notamment d'occupation, de menaces, d'agressions physiques, de tirs et d'affrontements armés. Ces attaques répétées ont probablement aggravé leur impact opérationnel, même si les attaques individuelles n'étaient pas toujours immédiatement invalidantes. Sur les 127 attaques, 36 (28,3 %) relevaient de plusieurs catégories, soulignant la complexité et la nature multidimensionnelle de la violence à laquelle sont confrontés les établissements de santé. Ces schémas cumulatifs et complexes sont difficiles à quantifier à partir des seules données sanitaires de routine. Des recherches qualitatives complémentaires pourraient contribuer à expliquer pourquoi certaines attaques entraînent des perturbations plus importantes que d'autres et comment des facteurs tels que l'éloignement, les chaînes d'approvisionnement ou le soutien communautaire influencent la réponse et le rétablissement du système de santé.

Bien que les types d'attaques les plus courants n'impliquaient pas de violences physiques ou sexuelles, une part importante (23,3 %) en a été affectée. Nous avons notamment identifié 13 cas de meurtre, un résultat alarmant étant donné que ces décès n'ont peut-être pas été enregistrés par les systèmes de surveillance existants. Les taux de violences personnelles étaient plus élevés que ceux documentés en Ukraine au cours des 18 premiers mois du conflit : 7,2 % des attaques de notre étude impliquaient des meurtres (contre 2 % en Ukraine) et 15,9 % impliquaient d'autres formes d'agression (contre 4 % ayant entraîné des blessures en Ukraine)^14^.

Nos résultats suggèrent qu'en RCA, les effets des attaques sur les soins de santé ont été variables. Certaines attaques ont entraîné la fermeture complète des établissements, d'autres ont affecté des services spécifiques, et certaines ont eu un impact minimal ou de courte durée. La variation de l'impact est illustrée par des cas comme celui du centre de santé de Tiringoulou, qui a connu une perturbation minimale de ses services grâce à des facteurs atténuants tels que la proximité d'une piste d'atterrissage, qui a assuré une chaîne d'approvisionnement stable. En revanche, l’insécurité et le manque d’accès ont probablement retardé la reprise des services dans les zones plus reculées.

Des différences de niveau de service ont également été observées. Les accouchements en établissement ont probablement été moins affectés, car les naissances ne peuvent être reportées, les soins obstétricaux dépendent moins des chaînes d'approvisionnement, les compétences des prestataires conservent leur valeur même avec des ressources limitées et les maternités sont souvent dotées de personnel communautaire formé. Ces résultats concordent avec les recherches menées en Syrie^20^ et en Ouganda^12^, qui ont montré que les accouchements assistés peuvent être moins perturbés par la violence que d'autres services de santé et peuvent même augmenter en période d'insécurité.

En revanche, les services de vaccination ont été particulièrement vulnérables, subissant des baisses importantes dues aux perturbations de la chaîne d'approvisionnement ou à la perte de personnel, suivies de pics d'activité une fois les services rétablis. Les attaques ont souvent entraîné un renforcement des protocoles de sécurité pour les acteurs de la santé, notamment des restrictions de circulation routière, ce qui a empêché la livraison de vaccins et de fournitures.

#### Construire des systèmes de santé résilients

Bien que cette étude n'ait pas évalué l'efficacité des mesures de mitigation, les résultats suggèrent que les approches rigides et uniformes ont peu de chances de fonctionner. Les réponses doivent être flexibles et adaptables. Étant donné que le pillage est courant, il est essentiel de renforcer les chaînes d'approvisionnement pour protéger les programmes de vaccination ainsi que les autres services. C'est particulièrement urgent en RCA, où la couverture vaccinale de routine reste précaire. En 2024, la couverture vaccinale nationale contre la rougeole était estimée à 25,5 %^38^. Une évaluation réalisée en 2021 à Vakaga a révélé que 13 des 21 établissements ne disposaient pas d'un entrepôt de vaccins fonctionnel, les ruptures de stock étant exacerbées par le recours à des établissements voisins ou à des cliniques mobiles. L'insécurité perturbe également la circulation des fournitures, ce qui rend la couverture vaccinale difficile, même en l'absence d'attaques directes^13^.

Des plans de mitigation adaptés peuvent contribuer à réduire les conséquences à long terme des attaques sur les services de santé. Les conclusions des entretiens approfondis soulignent le rôle des communautés locales et du personnel de santé dans le maintien des services après les attaques^6^. Les organisations humanitaires et les donateurs devraient donner la priorité à un financement flexible et à des initiatives de renforcement des capacités afin d'améliorer la résilience locale. Malgré des attaques consécutives, notamment des meurtres et des pillages, le poste de santé d'Irabanda est resté pleinement opérationnel, à l'exception de la vaccination, grâce au dévouement de son personnel et de sa communauté. Avant l'attaque, l'établissement était soutenu par un projet de gestion intégrée des cas communautaires qui mettait l'accent sur le renforcement des capacités locales. En outre, l'éloignement de l'établissement a laissé peu d'alternatives à la population, ce qui a pu motiver le personnel à poursuivre ses activités malgré les risques sécuritaires. L'implication des acteurs locaux dans les efforts d'atténuation et de réponse améliorera leur succès.

Pour être efficaces, les réponses doivent tenir compte du type d'attaque et des services touchés. Le renforcement des chaînes d'approvisionnement en vaccins pourrait aider à maintenir les taux de vaccination, tandis que l'amélioration des soins obstétriques d'urgence permettra de mieux répondre aux perturbations des services maternels. D'autres approches visant à renforcer la résilience du système de santé peuvent inclure l'investissement dans l'infrastructure de la chaîne du froid pour chaque établissement afin de réduire la dépendance à l'égard des approvisionnements externes, l'expansion des initiatives de santé communautaire et l'intégration d'un paquet minimum de services pour la vaccination dans les zones éloignées ou peu sûres. Les cliniques mobiles peuvent servir de solutions temporaires lorsque les services sont perturbés. À Irabanda, nous avons observé un pic de vaccinations plus d'un an après la suspension des services grâce au soutien des cliniques mobiles. En fin, le plaidoyer, la médiation et le dialogue avec les groupes armés sont essentiels pour protéger le personnel, les patients et les soignants, et pour garantir l'accès aux soins dans les établissements et pour les services de proximité^39^.

### Données sur les attaques

La collecte de données sur les attaques dans les zones de conflit est difficile, ce qui conduit souvent à des rapports incomplets et incohérents. Nous avons combiné des sources primaires et secondaires, notamment des rapports internes d’IMC, des documents d’Insecurity Insight, des publications de Radio Ndeke Luka et une base de données anonyme, pour créer un ensemble de données plus complet. Des recherches menées en Syrie ont également montré que la combinaison des sources de données améliorait la documentation des attaques^34^. Nous avons triangulé les sources, mené plusieurs entretiens par attaque et extrait systématiquement des variables entre les sources pour améliorer l’exactitude, l’exhaustivité et la fiabilité des données.

L’accès à des données de santé de qualité au niveau des établissements est une condition préalable à l’analyse quantitative de l’utilisation des services. Nous avons utilisé des données agrégées hebdomadaires provenant du système d’information sanitaire d’IMC. Malgré des ressources limitées, nous avons documenté 127 attaques dans trois préfectures de 2016 à 2020, contre 80 attaques enregistrées à l’échelle nationale par l’OMS au cours de la même période^22^. Seulement 13 % des attaques correspondent à plusieurs sources, ce qui souligne l’importance d’utiliser plusieurs sources et différentes méthodes de collecte de données pour obtenir une image complète du paysage des attaques. La plupart des attaques n’ont été identifiées que dans les données d’incidents de l’IMC, qui, comme d’autres dossiers de prestataires de services, sont rarement partagées en externe.

Certaines ONG internationales ont fait des efforts pour améliorer la transparence des données. Cependant, l’absence de partage systématique des données indique des problèmes structurels majeurs dans le signalement, ce qui conduit à une grave sous-estimation de l’ampleur de la violence contre les soins de santé. En outre, l’accès aux données du SIS nécessite des partenariats avec des ONG locales ou internationales ou des équipes de santé de district. Dans les situations de conflit, les SIS sont souvent perturbés^41^ et les acteurs peuvent être réticents à partager les données^42^, introduisant des biais basés sur l’accès géographique ou les caractéristiques de la population.

Sans rapports précis, il reste difficile de suivre les attaques, d’allouer des ressources et d’élaborer des stratégies de mitigation. Parada et al. ont comparé deux ensembles de données d'attaques contre les soins de santé, dérivés d'attaques publiquement signalées contre les soins de santé, et n'ont trouvé qu'un chevauchement de 13 %, soulignant la sous-déclaration des attaques et l'incohérence dans les ensembles de données résultant de la variation des sources de données et des processus de conservation^43^. Une étude de cadrage a identifié la collecte systématique de données comme une recommandation essentielle pour répondre aux attaques^44^. Notre étude renforce cette recommandation; alors que nous avons identifié 127 attaques dans les zones d’étude, Insecurity Insight n’a enregistré aucun événement dans la Vakaga et 32 ​​événements dans les préfectures de la Ouaka et de la Haute-Kotto au cours de la même période, dont la plupart se sont produits en dehors des zones de captage des installations soutenues par l’IMC.

### Améliorer les mécanismes de signalement des attaques contre les soins de santé

Le *Système de surveillance des attaques contre les soins de santé* (SSA) de l’OMS a été créé pour documenter de manière exhaustive les attaques, mais a été critiqué à juste titre pour ne pas avoir atteint son objectif^45,46^. Le nombre d'attaques identifiées dans notre étude dépasse largement celui rapporté par la SSA ; plus précisément, la SSA a signalé 80 attaques entre 2016 et 2020 dans l'ensemble du pays^37^. Une recherche récente menée en Ukraine sur les attaques contre les soins de santé, qui s'appuyait uniquement sur la SSA de l'OMS, a révélé des niveaux de violence physique inférieurs à ceux de notre étude^14^. Cela peut être dû à des différences de contexte, ou cela peut être une preuve supplémentaire des lacunes de la SSA dans la documentation exhaustive des attaques. Nos conclusions soulignent la nécessité d'évaluer et d'améliorer la méthodologie de la SSA, notamment en renforçant la sensibilisation et la communication avec les ONG internationales et locales, les groupes de la société civile et les prestataires de santé. L'OMS doit adopter une approche plus transparente et coopérative en matière de collecte et de communication des données, une approche qui ne compromet pas la confidentialité ou la sécurité, mais qui facilite un partage et une responsabilisation plus larges^47^.

Il est essentiel d’établir un mécanisme mondial fonctionnel, fiable et sécurisé pour documenter les attaques contre les soins de santé. Le partage des données et la transparence peuvent être renforcés par des accords formels signés entre les ONG, les donateurs et des acteurs globales comme l’OMS. Des rapports inadéquats créent une fausse perception dangereuse selon laquelle les attaques sont peu fréquentes, mineures ou n’ont pas d’effets significatifs à long terme.

Les gouvernements, les acteurs humanitaires, les chercheurs, les communautés et les donateurs doivent collaborer pour améliorer la collecte de données et partager les rapports d’incidents afin de comprendre pleinement l’impact de ces attaques. La triangulation des données a permis d’obtenir une image plus complète de leur ampleur et de leur étendue. La surveillance en temps réel, la fréquence accrue des rapports et les directives de non-déclaration amélioreront également l’exactitude et l’exhaustivité des données, permettant ainsi une analyse plus solide. L’intégration des données sur les attaques dans des systèmes d’information de santé numériques comme DHIS2^48^ pourrait faciliter un meilleur suivi, la collecte des données et analyse plus durables et continues, une réponse plus rapide et une prise de décision éclairée. Le renforcement des mécanismes mondiaux de signalement, notamment de la SSA, est essentiel pour comprendre le véritable coût de ces attaques.

Malgré les difficultés que pose la conduite de recherches dans des contextes fragiles, les résultats soulignent la nécessité impérieuse de réaliser des évaluations solides et fondées sur des données pour quantifier l’impact des attaques sur les soins de santé. L’élargissement de ces efforts contribuera à renforcer les mesures de protection des systèmes de santé dans les zones de conflit et à améliorer la défense des droits des populations touchées.

Les efforts visant à améliorer les mécanismes de signalement doivent être complétés par un plaidoyer visant à prévenir et à atténuer les impacts des attaques et à traduire les auteurs en justice. Le plaidoyer contre les violences visant les soins de santé doit être une priorité mondiale. Un plaidoyer plus fort est essentiel pour sensibiliser, exiger des comptes et garantir que la protection des soins de santé soit au cœur de la réponse humanitaire, de l'élaboration des politiques et de la sécurité sanitaire mondiale. Lorsque les attaques sont sous-déclarées ou ignorées, il devient difficile de mobiliser l'action, car le plaidoyer ne peut réussir sans preuves. Un meilleur signalement et un plaidoyer fort sont complémentaires et doivent aller de pair pour réduire la fréquence et l'impact des attaques. Sans efforts urgents et soutenus sur ces deux fronts, la normalisation des violences contre les soins de santé continuera de compromettre les résultats sanitaires et de violer les droits humains fondamentaux. De même, le plaidoyer est essentiel pour garantir la mise en place de mesures d'atténuation, notamment un soutien aux professionnels de santé et aux communautés victimes d'attaques.

### Nouvelle application des méthodes existantes

Cette étude a appliqué le SCI et l’analyse de survie pour étudier l’impact des attaques sur les soins de santé. Bien que ces méthodes soient bien établies en santé publique, leur application pour quantifier les effets de la violence sur les soins de santé est relativement nouvelle. Le SCI nous a permis de mesurer les changements dans l’utilisation des soins de santé après une attaque sur une période d’un an, en comparant les résultats observés à des scénarios contrefactuels sans attaques. L’analyse de survie a lié les perturbations des soins de santé à des maladies évitables par la vaccination comme la rougeole. Cette approche est particulièrement utile dans les contextes fragiles où les soins alternatifs ne sont pas disponibles et où les perturbations des services peuvent entraîner des épidémies, une augmentation de la mortalité maternelle et néonatale et une incidence plus élevée de la malnutrition.

L’utilisation d’une approche mixte a permis de mieux comprendre les impacts. L’analyse quantitative a mesuré objectivement la gravité et l’étendue des perturbations, tandis que les données qualitatives ont contextualisé les résultats, identifiant les facteurs qui ont continué à influencer l’utilisation des soins de santé après les attaques. Cette intégration a mis en évidence les impacts directs et les stratégies de réponse, en capturant à la fois les tendances statistiques et les expériences humaines. L’application de méthodes de santé publique établies dans ce contexte a démontré comment les attaques peuvent affecter différents services de santé, renforçant la nécessité d’approches ciblées et fondées sur les données pour la protection, l’atténuation et le rétablissement des soins de santé. La compréhension de ces impacts permet d’adapter les interventions à la nature et à l’ampleur des perturbations, améliorant ainsi la réponse dans les contextes touchés par les conflits.

Importance de l'évaluation quantitative des attaques contre les soins de santé

Ce travail décrit le volet quantitatif d'une étude à méthodes mixtes portant sur l'impact des attaques sur les soins de santé en RCA. Comprendre ces impacts est crucial pour préserver la santé et les droits humains, réduire les souffrances et prévenir les décès évitables, en particulier dans les contextes fragiles où l'accès et la qualité des soins sont déjà fortement limités. Les études qualitatives ont historiquement dominé la recherche sur les attaques contre les soins de santé^2-8^, en raison des difficultés à obtenir des données fiables sur les établissements de santé dans les zones de conflit^49^ et à isoler les effets sur les soins de santé de la dynamique plus large du conflit. Si la recherche qualitative fournit des informations précieuses, elle ne parvient pas à saisir toute l'ampleur et la variabilité de l'impact des attaques sur les systèmes de santé, l'utilisation des services et le bien-être de la population, en particulier à long terme.

À quelques exceptions près^9, 10^, les travaux quantitatifs sur l'impact des attaques contre les soins de santé se sont concentrés sur des contextes extérieurs à l'Afrique subsaharienne. Par exemple, en Syrie, Burbach et al. ont constaté que les attaques contre les soins de santé étaient significativement associées à une baisse des consultations ambulatoires et traumatologiques et des accouchements en établissement^13^. Ekzayez et al. ont constaté qu'en Syrie, la violence était associée à une légère baisse des consultations ambulatoires et prénatales, et à une augmentation des accouchements et des césariennes^20^. Cependant, le système de santé syrien d'avant le conflit était plus développé, ce qui rend les comparaisons directes difficiles. Les crises négligées comme celle de la RCA reçoivent le moins d'attention de la recherche, malgré des besoins parmi les plus importants et des lacunes de données parmi les plus importantes. Ce manque de connaissances et de données a de graves implications, car le manque de preuves empêche des interventions bien informées. En quantifiant les effets immédiats et à long terme des attaques en RCA, cette étude démontre comment les méthodes quantitatives, en particulier en conjonction avec des données qualitatives, peuvent améliorer la compréhension des coûts réels et différentiels des attaques contre les soins de santé – en particulier pour la population touchée – et renforcer la base de données probantes pour des interventions meilleures et ciblées, et améliorer la protection des soins de santé en période de conflit.

### Limites et défis

Nos résultats doivent être interprétés avec prudence en raison de plusieurs limites importantes. Les impacts observés peuvent être influencés par des facteurs non mesurés, notamment l’état des installations avant l’attaque, la situation géographique, la ruralité, l’accessibilité, la durée et la fréquence des attaques, la dotation en personnel, le financement et la dynamique locale. Nous n’avons pas pu contrôler l’insécurité environnante, les déplacements ou les perturbations socioéconomiques, ce qui limite notre capacité à définir des relations de cause à effet entre les attaques et les résultats en matière de soins de santé.

Nous avons également rencontré des difficultés méthodologiques importantes. La fréquence des attaques a rendu difficile la recherche d’installations de contrôle pour l’analyse SCI. A Vakaga, le regroupement des attaques en 2020 a limité notre capacité à évaluer les impacts à long terme. Les hypothèses SCI ont probablement été violées car les attaques successives ont perturbé la continuité des données. Des données incomplètes et incohérentes ont encore limité l’étude. L’absence de rapportage nul, les rapports peu fréquents et les périodes où l’IMC n’a pas soutenu certaines structures de santé ont probablement entraîné une sous-déclaration d’incidents mineurs, tels que des menaces verbales. L’absence de données ne permettait pas de savoir si le nombre zéro de cas reflétait la réalité ou un défaut de rapportage, bien que nous ayons utilisé des indicateurs indirects (par exemple, des données de consultation) pour combler les lacunes lorsque cela était possible. Ces incohérences ont probablement sous-estimé l’impact cumulé d’attaques plus petites et fréquentes et masqué la gravité des interruptions de service.

La qualité des données secondaires des établissements de santé utilisées dans cette étude a également posé des limites. Les systèmes d'information sanitaire de routine dans les zones de conflit peuvent souffrir d'incohérences dans les rapports, de lacunes dans la couverture et d'une vérification limitée, ce qui peut introduire des biais dans les rapports ou affecter la fiabilité des tendances d'utilisation des services au fil du temps. L’absence de données sur la date des attaques a obligé à regrouper les données par mois, ce qui a masqué les fluctuations à court terme de l’utilisation des soins de santé et réduit la précision de l’analyse SCI. De plus, l’incapacité à quantifier la gravité des attaques ou à tenir compte des attaques qui se chevauchent a rendu difficile l’isolement des effets individuels. Les attaques multiples ont probablement aggravé les perturbations, ce qui a compliqué l’analyse. Les entretiens avec des informateurs clés peuvent être sujets à un biais de rappel, en particulier pour des événements survenus plusieurs années auparavant, et les rapports peuvent être influencés par le rôle du répondant, ses expériences ou sa relation avec l’établissement.

Nous n'avons pas pu pleinement prendre en compte le soutien des prestataires privés, des agences des Nations Unies ou des ONG dans les préfectures en raison de l'absence de données complètes sur leur présence et leur couverture. Limiter l'analyse aux établissements soutenus par l'IMC a pu biaiser l'étude, car ces établissements peuvent différer des établissements ne bénéficiant pas d'un soutien externe ou des établissements privés. Les sites soutenus par l'IMC bénéficient probablement de systèmes de rapportage et de capacités opérationnelles améliorés et peuvent être plus ou moins susceptibles d'être ciblés, selon le contexte. Par conséquent, la généralisation de nos résultats à l'ensemble du système de santé pourrait être limitée.

L'étude n'a pas évalué la qualité des soins. Bien que les consultations des patients aient été enregistrées, on ignore si les prestataires qualifiés ont dispensé un traitement approprié. Cette limitation est particulièrement pertinente compte tenu de la forte augmentation observée de l'utilisation des services après certaines attaques, qui a coïncidé avec des pics de violence régionale. Ces augmentations pourraient refléter le déplacement de populations se rendant dans des établissements soutenus par l'IMC en raison d'attaques contre d'autres sites de soins de santé voisins non inclus dans cette étude. Ces augmentations pourraient avoir affecté la qualité des soins prodigués, un facteur non évalué ici mais reconnu par les auteurs de l'étude. Compte tenu de ces difficultés, nos résultats sous-estiment probablement l'ampleur réelle de l'impact des attaques sur les soins de santé.

De plus, l'absence de signaux quantitatifs forts de perturbation des services ne doit pas être interprétée comme une absence d'impact négatif. Ces effets pourraient être mieux appréhendés grâce au volet qualitatif complémentaire de l'étude, qui a exploré les points de vue des patients et des prestataires sur les conséquences des attaques.

Enfin, l'étude n'a pas pu inclure de mesures à l'échelle de la population, ce qui aurait renforcé l'analyse, en raison des difficultés inhérentes à la collecte de telles données dans des contextes de conflit comme le nord de la RCA. Par conséquent, les résultats ne reflètent peut-être pas pleinement l’impact des attaques sur les soins de santé sur la population dans son ensemble.

### Conclusions

Pour réduire l’impact des attaques sur les soins de santé, il faut mieux exploiter les données et s’engager politiquement. Il est essentiel de mettre en place un mécanisme mondial fonctionnel, fiable et sécurisé pour documenter les attaques contre les soins de santé. Le renforcement de la surveillance en temps réel, de l’intégration dans les systèmes de santé numériques et du partage des données améliorera les efforts de réponse et de plaidoyer. Les acteurs humanitaires, les gouvernements, les chercheurs et les donateurs doivent collaborer pour garantir une compréhension plus précise de l’ampleur et de l’impact des attaques. En outre, les réponses doivent être adaptées au type d’attaque et aux services concernés. Les stratégies d’atténuation et les efforts de réponse doivent avoir l’implication significative communautés touchées, car elles sont les premières et principales personnes à intervenir en cas d’attaque. Le plaidoyer, la médiation et le dialogue avec les groupes armés sont également essentiels pour garantir l’accès et la protection des professionnels de santé, des patients et des équipes mobiles.

La réponse à la crise de la violence contre les soins de santé exige un engagement ferme de tous les acteurs à respecter le caractère sacré de la vie et à faire respecter le droit international humanitaire. Nous appelons à une collaboration et une action mondiales urgentes pour protéger les services de santé dans les zones de conflit et préserver la vie des populations vulnérables. Ces résultats soulignent le besoin crucial d’évaluations solides et fondées sur des données concernant les attaques contre les soins de santé dans les zones de conflit et les crises négligées.

## References

1. Insecurity Insight. Critical Condition: Violence Against Health Care in Conflict. 2023.

2. Abbara A, Rayes D, Tappis H, Hamze M, Wais R, Alahmad H, et al. "Actually, the psychological wounds are more difficult than physical injuries:" a qualitative analysis of the impacts of attacks on health on the personal and professional lives of health workers in the Syrian conflict. Confl Health. 2023;17(1):48.

3. Elnakib S, Elaraby S, Othman F, BaSaleem H, Abdulghani AlShawafi NA, Saleh Al-Gawfi IA, et al. Providing care under extreme adversity: The impact of the Yemen conflict on the personal and professional lives of health workers. Soc Sci Med. 2021;272:113751.

4. Footer KHA, Clouse E, Rayes D, Sahloul Z, Rubenstein LS. Qualitative accounts from Syrian health professionals regarding violations of the right to health, including the use of chemical weapons, in opposition-held Syria. BMJ Open. 2018;8(8):e021096.

5. Haar RJ, Crawford K, Fast L, Win TH, Rubenstein L, Blanchet K, et al. "I will take part in the revolution with our people": a qualitative study of healthcare workers' experiences of violence and resistance after the 2021 Myanmar coup d'etat. Confl Health. 2024;18(1):52.

6. Kostandova N, OKeeffe J, Ali BB, Somse P, Mahieu A, Bingou OG, et al. "It's normal to be afraid": attacks on healthcare in Ouaka, Haute-Kotto, and Vakaga prefectures of the Central African Republic, 2016-2020. Confl Health. 2024;18(1):54.

7. Neuman M. "No patients, no problems" Exposure to risk of medical personnel working in MSF projects in Yemen's governorate of Amran. J Humanit Assist. 2014.

8. Singh NS, Redman B, Broussard G, DeCamp M, Rayes D, Ho LS, et al. 'We will never give up': a qualitative study of ethical challenges Syrian health workers face in situations of extreme violence. Disasters. 2022;46(2):301-28.

9. Druetz T, Browne L, Bicaba F, Mitchell MI, Bicaba A. Effects of terrorist attacks on access to maternal healthcare services: a national longitudinal study in Burkina Faso. BMJ Glob Health. 2020;5(9).

10. Badri R, Dawood I. The implications of the Sudan war on healthcare workers and facilities: a health system tragedy. Confl Health. 2024;18(1):22.

11. Chukwuma A, Ekhator-Mobayode UE. Armed conflict and maternal health care utilization: Evidence from the Boko Haram Insurgency in Nigeria. Soc Sci Med. 2019;226:104-12.

12. Namasivayam A, Arcos Gonzalez P, Castro Delgado R, Chi PC. The Effect of Armed Conflict on the Utilization of Maternal Health Services in Uganda: A Population-based Study. PLoS Curr. 2017;9.

13. Burbach R, Tappis H, Abbara A, Albaik A, Almhawish N, Rubenstein LS, et al. Quantifying the effects of attacks on health facilities on health service use in Northwest Syria: a case time series study from 2017 to 2019. BMJ Glob Health. 2024;9(9).

14. Kim HJ, Bruni E, Gorodetska G, Van den Bergh R, Bezer L, Artykutsa S, et al. Typology and implications of verified attacks on health care in Ukraine in the first 18 months of war. PLOS Glob Public Health. 2024;4(5):e0003064.

15. Gesesew H, Kebede H, Berhe K, Fauk N, Ward P. Perilous medicine in Tigray: a systematic review. Confl Health. 2023;17(1):26.

16. Chandini MA, Van den Bergh R, Agbor Junior AA, Willliam F, Obi AMM, Ngeha NC, et al. "It is because of the love for the job that we are still here": Mental health and psychosocial support among health care workers affected by attacks in the Northwest and Southwest regions of Cameroon. PLOS Glob Public Health. 2023;3(11):e0002422.

17. Crawford K, Florez T, Rodriguez M, Cirado L, Read R, Haar R. "There is a fear that you will be attacked just for the act of working in health": a survey of experiences of violence against healthcare in Colombia. Confl Health. 2023;17(1):51.

18. International Rescue Committee. Joint Health Staff Survey Protection of Health Care In Northeast Nigeria. 2022 Oct.

19. International Rescue Committee. Joint Health Staff Survey: Protection of Health Care South Sudan. 2024 Oct.

20. Ekzayez A, Alhaj Ahmad Y, Alhaleb H, Checchi F. The impact of armed conflict on utilisation of health services in north-west Syria: an observational study. Confl Health. 2021;15(1):91.

21. Insecurity Insight SHiC. Central African Republic: Violence Against Health Care in Conflict 2021. 2022.

22. Insecurity Insight. Central African Republic (CAF): Attacks on Aid Operations and Health Care 2024 [Available from: <https://data.humdata.org/dataset/car-violence-against-civilians-and-vital-civlian-facilities>.

23. United Nations Development Program. Human Development Report 2023/2024: Breaking the Gridlock. 2024.

24. United Nations Office for the Coordination of Humanitarian Affairs. Central African Republic Humanitarian Response Plan. 2023 2023 Jul 19.

25. United States Agency for International Development. Central African Republic – Complex Emergency Fact Sheet 1. 2023.

26. World Health Organization. Density of doctors (physicians) per 10 000 population 2024 [Available from: <https://data.who.int/indicators/i/CCCEBB2/217795A>.

27. World Health Organization. HeRAMS Central African Republic Baseline Report 2023: Operational status of the health system. 2023.

28. Researching the Impact of Attacks on Healthcare. The University of Manchester; [cited 2021 Mar 22]. Available from: riah.manchester.ac.uk/.

29. World Health Organization. Stopping Attacks on Health Care 2022 [Available from: <https://www.who.int/activities/stopping-attacks-on-health-care>.

30. Radio Ndeke Luka. Radio Ndeke Luka, Fondation Hirondelle 2024 [Available from: <https://www.radiondekeluka.org/>.

31. Wood SN. Generalized Additive Models: An Introduction with R: Chapman and Hall/CRC; 2017.

32. Fulcher IR, Boley EJ, Gopaluni A, Varney PF, Barnhart DA, Kulikowski N, et al. Syndromic surveillance using monthly aggregate health systems information data: methods with application to COVID-19 in Liberia. Int J Epidemiol. 2021;50(4):1091-102.

33. Ministère de la Santé et de la Population. Epidémie de rougeole en RCA: déclaration du Ministère de la Santé et de la Population. 2020.

34. Government of Central African Republic, United Nations Children's Fund, World Health Organization. Epidémie de rougeole en Centrafrique, Grade 2 - Rapport de Situation N°3 Semaine épidémiologique 8 (17/02/2020 au 23/02/2020) Mise à jour du 24 février 2020. 2020.

35. Therneau TM. A Package for Survival Analysis in R. 2024.

36. Sjoberg DD, Baillie M, Fruechtenicht C, Haesendonckx S, Treis T. ggsurvfit: Flexible Time-to-Event Figures. 2024.

37. World Health Organization. Surveillance System For Attacks on Healthcare (SSA) 2024 [cited 2024 Mar 27]. Available from: <https://extranet.who.int/ssa/LeftMenu/Index.aspx>.

38. Ministère de la Santé Et de la Population. Enquête Nationale De Couverture Vaccinale De Routine Chez Les Enfants De 6-24 Mois

République Centrafricaine, Décembre 2023 – Mars 2024. 2024.

39. Geneva Call. 2024 [Available from: <https://www.genevacall.org/>.

40. Haar RJ, Risko CB, Singh S, Rayes D, Albaik A, Alnajar M, et al. Determining the scope of attacks on health in four governorates of Syria in 2016: Results of a field surveillance program. PLoS Med. 2018;15(4):e1002559.

41. Truppa C, Yaacoub S, Valente M, Celentano G, Ragazzoni L, Saulnier D. Health systems resilience in fragile and conflict-affected settings: a systematic scoping review. Confl Health. 2024;18(1):2.

42. Shalash A, Abu-Rmeileh NME, Kelly D, Elmusharaf K. The need for standardised methods of data collection, sharing of data and agency coordination in humanitarian settings. BMJ Glob Health. 2022;7(Suppl 8).

43. Parada V, Fast L, Briody C, Wille C, Coninx R. Underestimating attacks: comparing two sources of publicly-available data about attacks on health care in 2017. Conflict and Health. 2023;17(1):3.

44. Afzal MH, Jafar AJN. A scoping review of the wider and long-term impacts of attacks on healthcare in conflict zones. Med Confl Surviv. 2019;35(1):43-64.

45. Meier BM, Rice H, Bandara S. Monitoring Attacks on Health Care as a Basis to Facilitate Accountability for Human Rights Violations. Health Hum Rights. 2021;23(1):55-70.

46. Dadi AF, Mersha TB. WHO's surveillance system for attacks on health care is failing Ethiopia. The Lancet. 2022;399(10331):1225-6.

47. Haar R, Sirkin S. Strengthening Data to Protect Healthcare in Conflict Zones. International Peace Institute; 2022.

48. DHIS-2. In Action 2024 [Available from: <https://dhis2.org/in-action/>.

49. Haar RJ, Read R, Fast L, Blanchet K, Rinaldi S, Taithe B, et al. Violence against healthcare in conflict: a systematic review of the literature and agenda for future research. Confl Health. 2021;15(1):37.

### Appendix 2. Interrupted time series analysis

Summary of facilities included in ITS

| Prefecture | Facility | Date of first attack | Type of attack | Indicators included | Control considered? |
| --- | --- | --- | --- | --- | --- |
| Vakaga | CS Tiringoulou | 2018-05 | Pillage | Consultations, ANC1, Deliveries, MCV1, Hospitalizations | Yes – CS Sikikide |
| Ouaka | PS Madomale | 2017-10 | Pillage, agression, occupation, tuerie | Consultations, ANC1, MCV1 | Yes – PS Siou |
| Haute-Kotto | PS Boungou | 2017-07 | Tuerie | Consultations, ANC1, Deliveries | Yes – CS Bornou (PS Dangbatro pillaged) |
|  | PS Daba | 2017-09 | Pillage | Consultations, ANC1, Deliveries | No – Both PS Iramou and PS Issa Mazengue experienced attacks |
|  | PS Irabanda | 2018-08 | Pillage | Consultations, ANC1, Deliveries, MCV1 | No – PS Konengbe experienced attack |
|  | PS Issa Mazengue | 2017-09 | Pillage | Consultations, ANC1, Deliveries | No – both PS Daba and PS Iramou expereinced attacks |
|  | PS Konengbe | 2018-08 | Pillage | Consultations, ANC1, Deliveries | No – PS Irabanda experienced attacks |
|  | PS Ngoubi | 2017-11 | Pillage | Consultations, ANC1, Deliveries |  |

**Results of interrupted time series for PS Madomale, PS Konengbe, PS Issa Mazengue, PS Daba, and PS Boungou**

- **PS Madomale: Pillage on 2017-10**


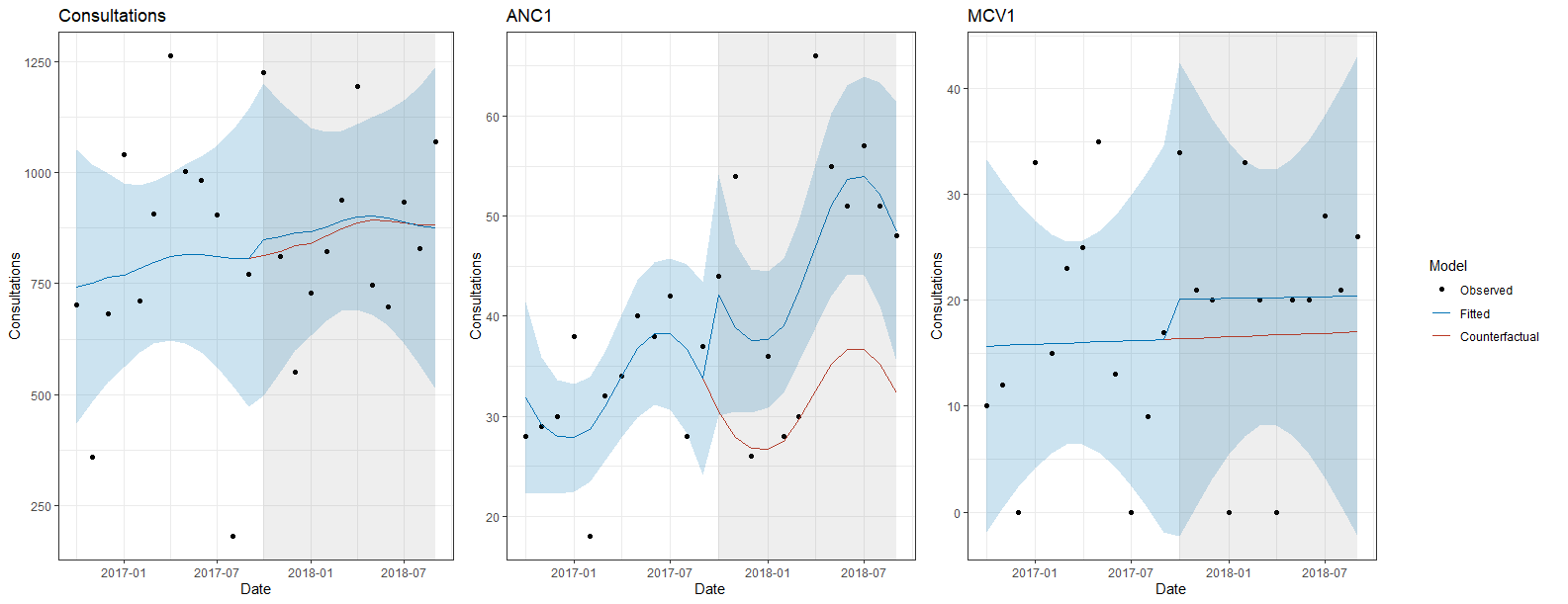


- **PS Konengbe: Pillage 2018-08**


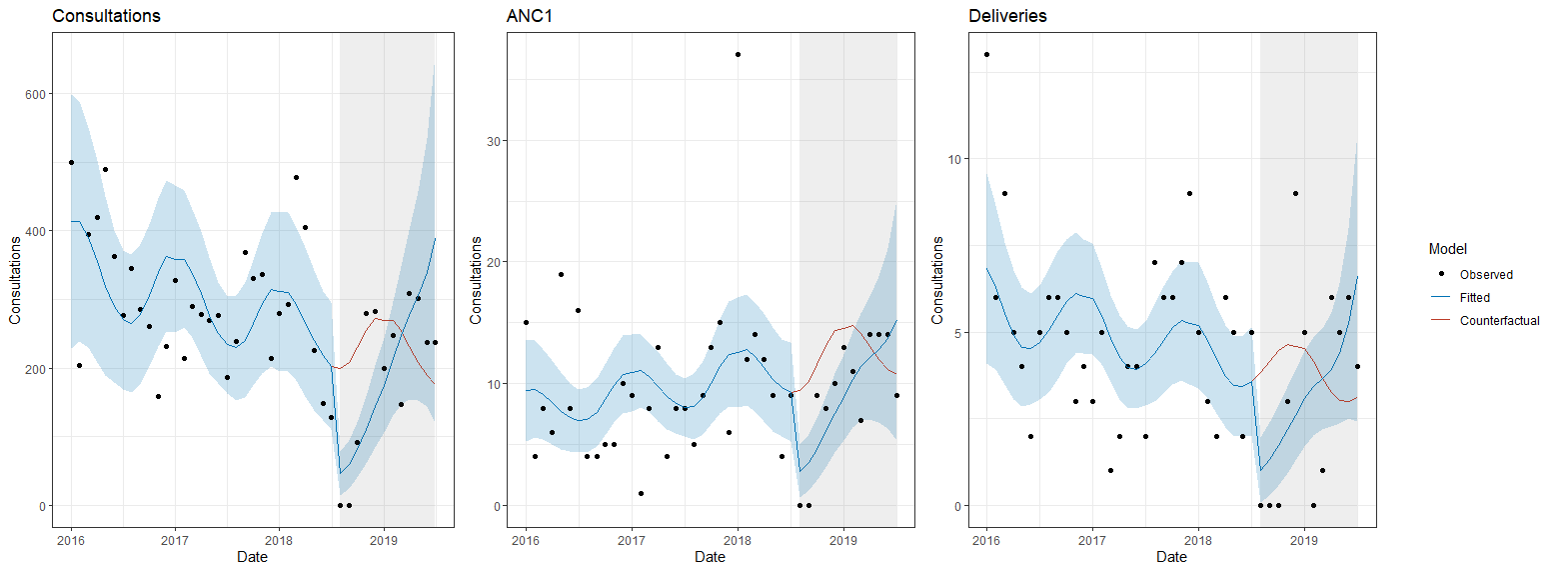


- **PS Issa Mazengue:** **Pillage 2017-09**
-
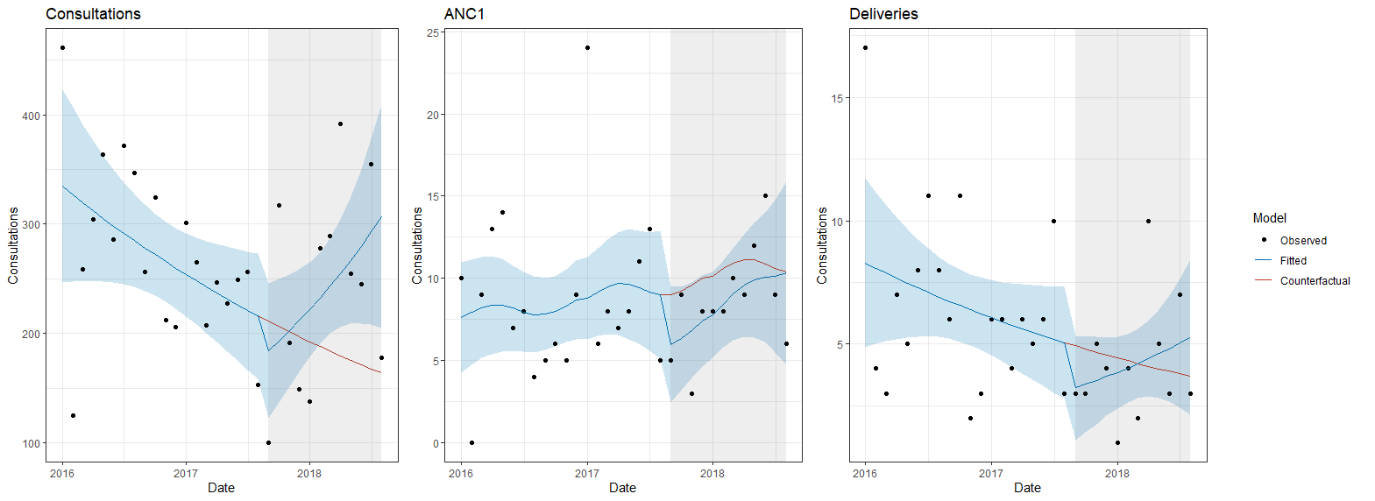

- **PS Daba:** Pillage 2017-09
-
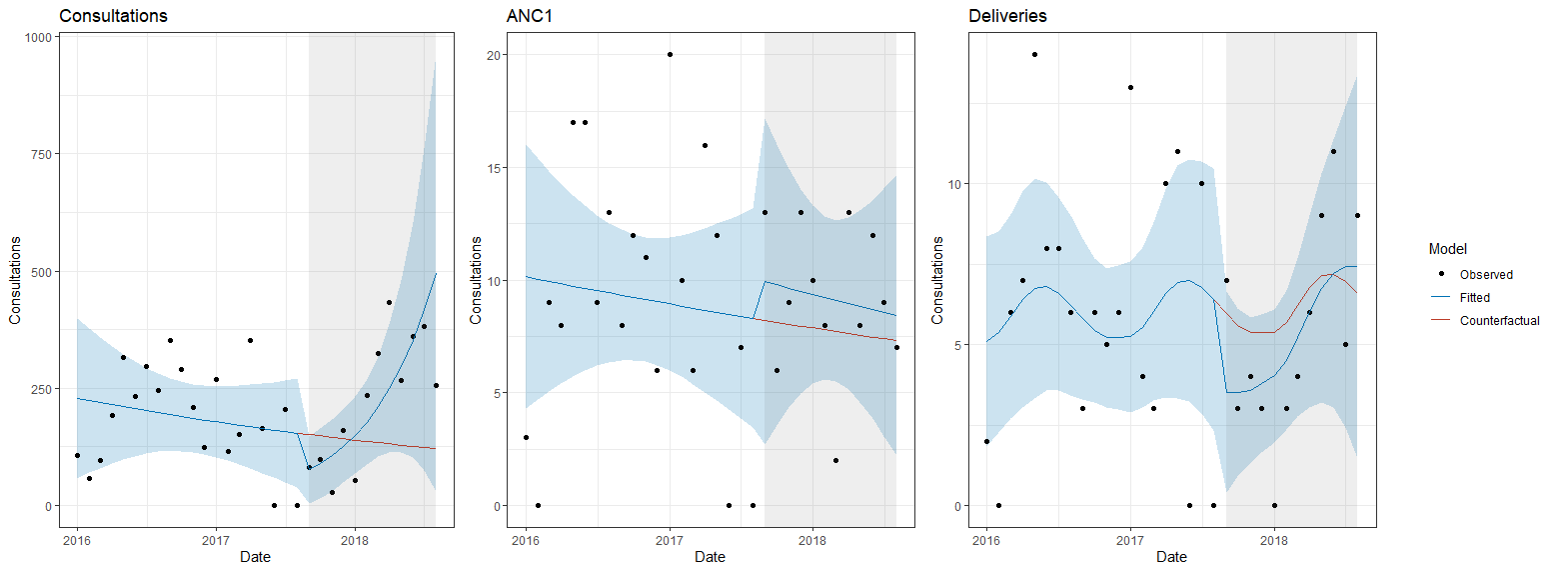

- **PS Boungou:** Multiple attacks, first is murder on 2017-07


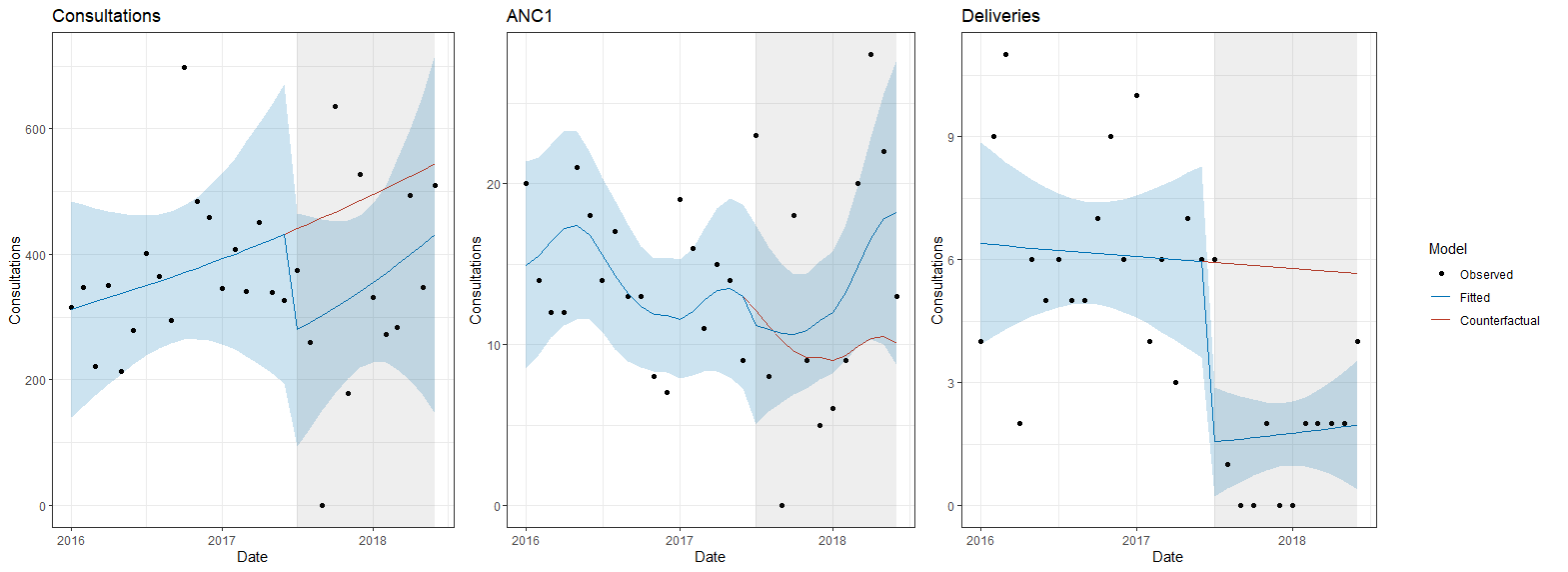


*Interrupted time series analysis with control facilities*

**CS Madomale w/control**


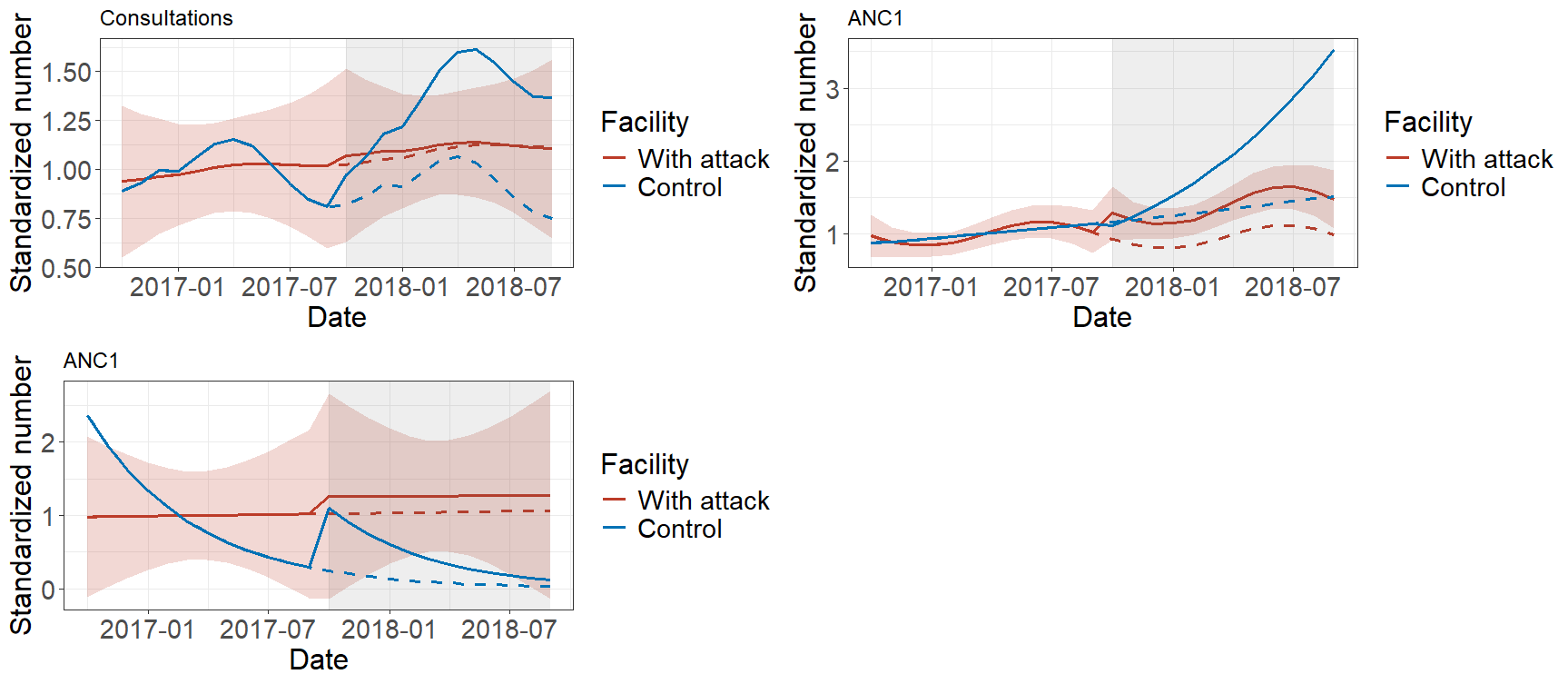


**PS Tiringoulou w/control**


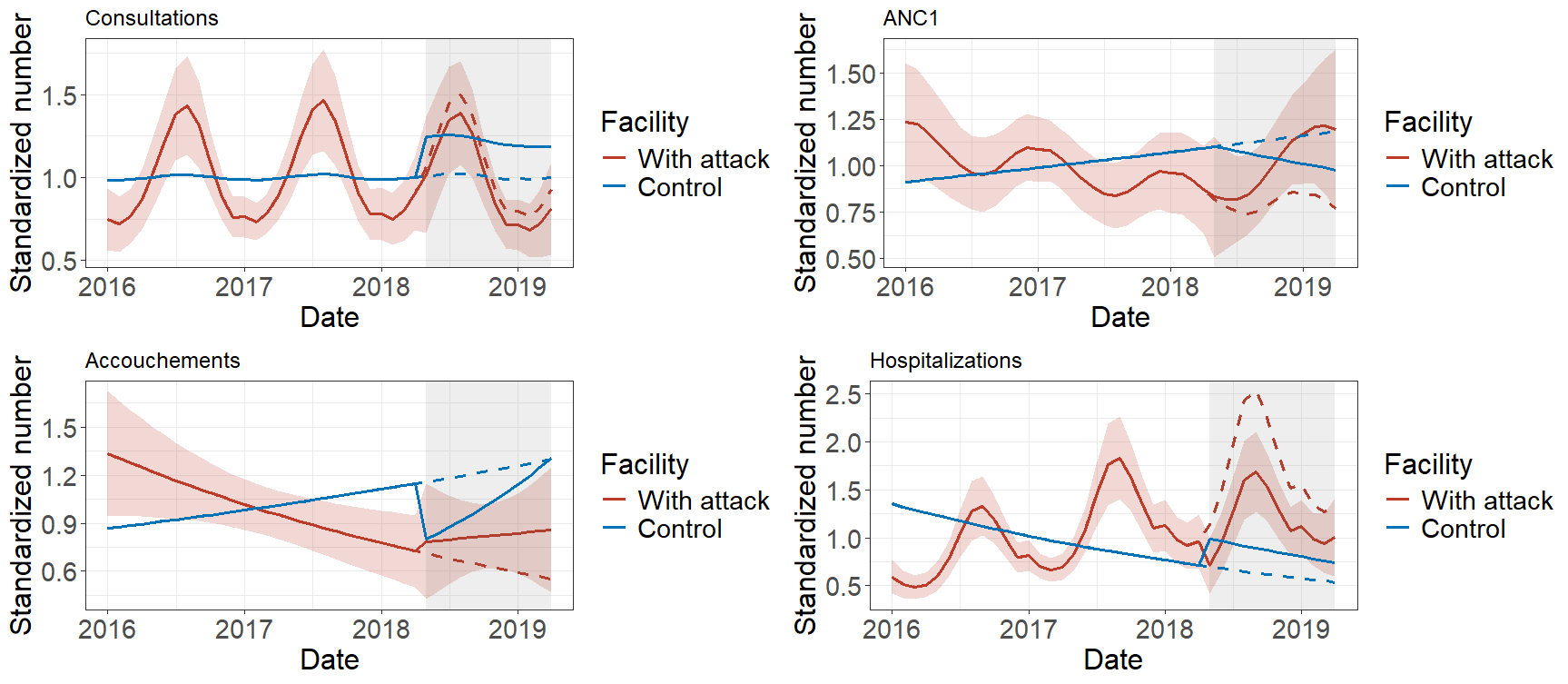


**Figure S1. Swimmer plot for time from first reported measles case in Vakaga to time to case in each of the health facilities.**


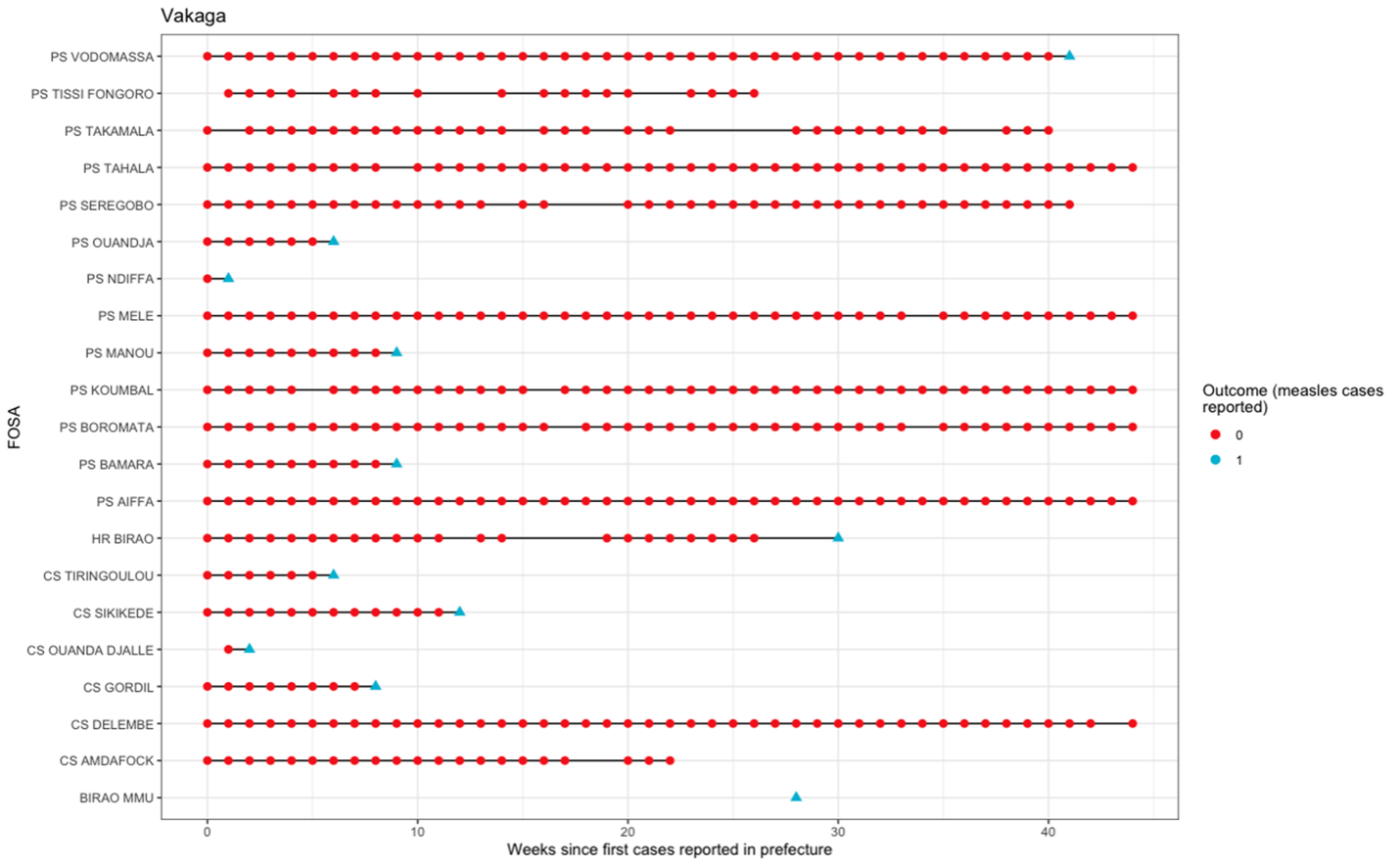

Supplement: Supplementary file 1 — Supplementary Material 1 [file 13031_2025_686_MOESM1_ESM.docx]
